# Multifunctional Three-Dimensional Porous MXene-Based Film with Superior Electromagnetic Wave Absorption and Flexible Electronics Performance

**DOI:** 10.1007/s40820-025-02034-2

**Published:** 2026-01-05

**Authors:** Li Chang, Xinci Zhang, Tingting Liu, Benyi Li, Ying Ji, Gongming Sun, Ziming Wang, Xitian Zhang, Maosheng Cao, Lin Li

**Affiliations:** 1 Key Laboratory for Photonic and Electronic Bandgap Materials, Ministry of Education, School of Physics & Electronic Engineering, Harbin Normal University, Harbin, 150025 People’s Republic of China; 2 School of Materials Science and Engineering, Beijing Institute of Technology, Beijing, 100081 People’s Republic of China

**Keywords:** 3D porous MXene films, Ni nanoparticles, Electromagnetic wave absorption, Interfacial polarization, Multifunctional sensing

## Abstract

The development of multifunctional electromagnetic wave-absorbing materials is essential for next-generation flexible electronics and intelligent protection systems. Herein, a novel three-dimensional porous MXene-based film integrated with metallic nickel nanoparticles (Ni-PMF) is designed and synthesized with the potential to address the urgent need for multifunctional electromagnetic wave-absorbing materials in next-generation intelligent systems. By using polystyrene spheres as sacrificial templates, a hierarchical porous architecture is constructed to prevent MXene nanosheet restacking, extend electromagnetic wave propagation paths, and optimize impedance matching. Simultaneously, uniformly distributed Ni nanoparticles introduce abundant heterogeneous interfaces, enhancing interfacial polarization and magnetic loss, which significantly improve electromagnetic wave attenuation. The Ni-PMF film achieves a minimum reflection loss of –64.7 dB and a broad effective absorption bandwidth of 7.2 GHz, covering the full Ku-band and outperforming most reported MXene thin film absorbers. In addition to superior electromagnetic wave absorption, the film demonstrates excellent electrothermal conversion and flexible strain-sensing capabilities, enabling integrated protection and real-time sensing functions. This multifunctional material offers promising potential for next-generation smart flexible electronic systems.

**Supplementary Information:**

The online version contains supplementary material available at 10.1007/s40820-025-02034-2.

## Highlights

- A multifunctional three-dimensional porous MXene-based film is fabricated, featuring a hierarchical porous structure that prevents nanosheet restacking and optimizes impedance matching.
- The MXene-based film integrated with metallic nickel nanoparticles (Ni-PMF) film with a wide effective bandwidth of 7.2 GHz, fully covering the Ku-band and surpassing most reported MXene-based film absorbers. Simultaneously, the Ni-PMF exhibits excellent electrothermal conversion and flexible strain-sensing capabilities.
- The Ni-PMF film integrates an electromagnetic attenuation mechanism, particularly abundant heterogeneous interfaces, thereby enhancing interfacial polarization and magnetic loss.

**Supplementary Information:**

The online version contains supplementary material available at 10.1007/s40820-025-02034-2.

## Graphical abstract

**Figure:**
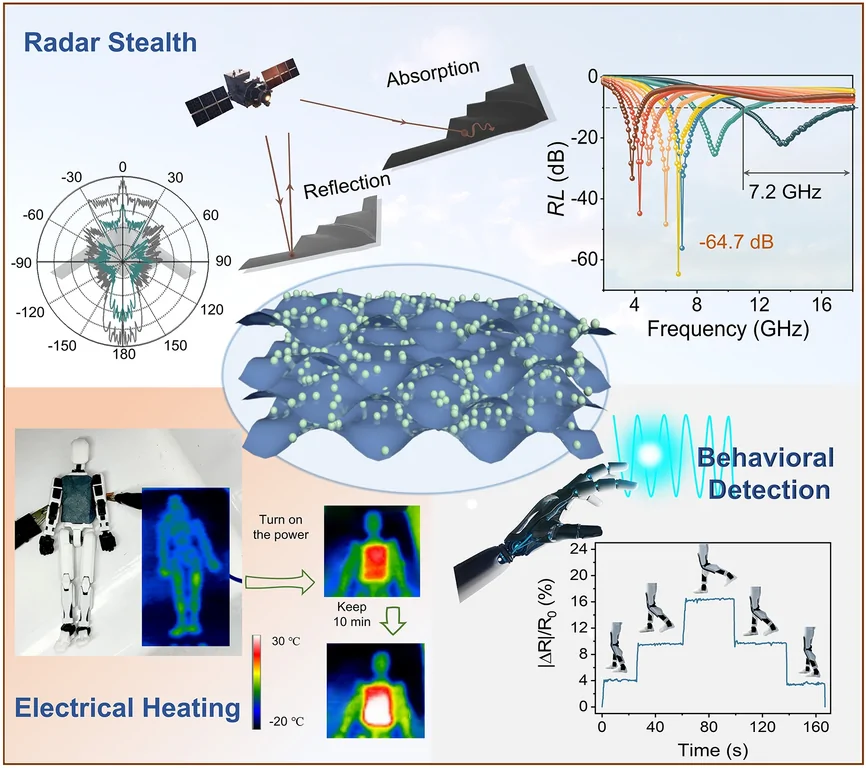

## Introduction

The rapid development of modern electronics and wireless communication technologies has brought unprecedented convenience to daily life. However, it has also led to the proliferation of electromagnetic (EM) pollution, which poses significant risks to the performance of sensitive electronic devices and potentially impacts human health [1]. Consequently, the development of efficient, thin, lightweight, and flexible electromagnetic wave (EMW)-absorbing materials has become an important research direction in the field of materials science. Compared with traditional powder materials, thin film materials exhibit superior conformability, low areal density, and excellent processability, making them ideal for applications in wearable electronics, aerospace, stealth technologies, and next-generation flexible electronic systems [2]. Furthermore, thin films allow for facile structural design and interfacial manipulation, enabling the construction of hierarchical architectures that enhance EMW dissipation and outstanding absorption properties through multiple scattering, reflection, and polarization mechanisms. At present, extensive research has focused on the development of high performance EMW-absorbing films to address the diverse and evolving requirements of next-generation intelligent electronic systems.

MXene, a rapidly emerging class of two-dimensional (2D) transition metal carbides and nitrides, has become one of the most promising materials for EMW absorption applications [3]. Benefiting from its excellent electrical conductivity, large specific surface area, tunable surface terminations, and layered structure, MXene exhibits versatile EMW attenuation capabilities, including dielectric loss and interface polarization [4]. Particularly, MXene films assembled by solution processing techniques can form flexible and conductive networks that facilitate the propagation and attenuation of EMWs. However, MXene-based materials still face several critical challenges that limit their EMW absorption performance. One of the major issues is the inherent tendency of MXene nanosheets to restack during the formation process of film, resulting in a densely packed structure with limited internal interfaces and reduced effective attenuation paths for incident EMWs [5, 6]. Moreover, the high electrical conductivity of MXene often leads to impedance mismatch, causing most EMWs to be reflected rather than absorbed [7, 8]. These drawbacks highlight the urgent need of developing structural engineering strategies to overcome the stacking issue and optimize the internal architecture of MXene films. To address this problem, introducing hierarchical porosity into MXene films has proven to be an effective strategy [9, 10]. Controlled microporous and mesoporous structures can be created within MXene matrices by using sacrificial templates such as polystyrene (PS) spheres [11]. This approach not only alleviates the stacking of 2D nanosheets but also introduces abundant internal voids that promote multiple scattering of EMWs, prolonging their propagation paths and enhancing absorption performance [12, 13]. Furthermore, the porous architecture facilitates better impedance matching by reducing the effective permittivity of the composite, thus allowing more EMW energy to penetrate into the material rather than being directly reflected. However, porosity alone is not sufficient to fully optimize EMW absorption; more sophisticated design strategies are necessary to achieve performance enhancements.

In addition to structural engineering, interface engineering has emerged as a crucial strategy for enhancing EMW absorption in functional materials [14]. By introducing magnetic nanoparticles into the MXene matrix to construct heterogeneous interfaces, multiple loss mechanisms can be effectively activated, thereby enhancing dielectric and magnetic losses and optimizing impedance matching [15, 16]. Due to the quasi-metallic nature of MXene, the interface formed with magnetic nanoparticles primarily resembles a metal–metal contact. Nevertheless, differences in work function combined with surface terminations such as –OH, –F, and –O induce localized charge accumulation at these interfaces [17]. This results in pronounced dipolar polarization under alternating EM fields, significantly boosting dielectric loss. Concurrently, magnetic nanoparticles contribute to magnetic loss through resonance and eddy current effects [18]. The synergy of dielectric and magnetic losses can broaden the absorption bandwidth and enhances attenuation efficiency [19]. Moreover, the magnetic nanoparticle/MXene interface may exhibit Schottky-like characteristics at defect-rich or surface functionalized regions of MXene. These localized potential barriers facilitate dynamic charge trapping and release, further promoting interfacial polarization and energy dissipation [20]. Although these promising strategies have been developed, it is still challenging to realizing MXene-based thin films with high EMW absorption performance.

It is noteworthy that current research on EM functional materials is increasingly shifting toward multifunctional integration [21, 22]. Intelligent materials that simultaneously possess EMW absorption and sensing capabilities have attracted considerable attention [23]. This is particularly important for applications in wearable electronics, smart monitoring systems, and flexible electronic devices, where materials are expected not only to provide effective EMW absorption but also to respond to various environmental stimuli, such as strain, electrical signals, and temperature changes [24]. Achieving “protection + perception” through multi-field synergistic response has become a key goal. Therefore, the development of multifunctional EM materials represents a critical technological pathway for enabling next-generation intelligent electronic systems.

Based on the above considerations, this work proposes a novel strategy for constructing a three-dimensional (3D) porous MXene-based film integrated with metallic Ni (3D Ni-PMF), which achieves multifunctional performance within a single material system. The 3D Ni-PMF combines structural porosity and interfacial engineering in a synergistic manner. By employing PS spheres as sacrificial templates, a well-defined hierarchical porous architecture is fabricated, which more effectively alleviates nanosheet restacking and extends EMW propagation pathways compared with random foams or layered composites. Simultaneously, Ni nanoparticles are incorporated into the porous MXene framework, producing abundant heterogeneous interfaces that not only induce strong interfacial polarization but also introduce additional magnetic losses [25]. This dual strategy markedly improves impedance matching and enables efficient EM energy dissipation [26, 27]. As a result, the 3D Ni-PMF achieves an exceptional minimum reflection loss (*RL*_min_) of –64.7 dB, along with a broad effective absorption bandwidth (EAB_10_, defined as *RL* ≤ –10 dB) of 7.2 GHz, successfully covering the full Ku-band. Importantly, its EM absorption performance surpasses most previously reported MXene-based thin film absorbers. Moreover, the 3D Ni-PMF exhibits excellent electrothermal conversion and flexible strain-sensing capability, thereby realizing integrated “protection + perception.” This multifunctional design provides a new material platform for flexible electronics, protective armors, and intelligent sensing systems, offering promising solutions for next-generation smart applications.

## Experimental Section

### Materials

Nickel chloride hexahydrate (NiCl_2_·6H_2_O), lithium fluoride (LiF), lithium chloride (LiCl), and sodium hydroxide (NaOH) were purchased from Shanghai Macklin Biochemical Co., Ltd. Kevlar® 49 thread was obtained from DuPont (USA). Hydrochloric acid (HCl, 37%) was supplied by Sinopharm Group Co., Ltd. Styrene, dimethyl sulfoxide (DMSO), sodium borohydride (NaBH_4_), potassium hydroxide (KOH), and potassium persulfate (K_2_S_2_O_8_) were purchased from Aladdin Chemical Reagent Co., Ltd. (Shanghai, China). Titanium aluminum carbide (Ti_3_AlC_2_, 200 mesh) powders were provided by Laizhou Kai Kai Ceramic Materials Co., Ltd. All chemicals were used as received without further purification.

### Synthesis of MXene (Ti_3_C_2_Tₓ) Nanosheets

MXene (Ti_3_C_2_T_X_) was synthesized by selective etching of Al layers from Ti_3_AlC_2_. Briefly, 1.56 g of LiF was dissolved in 20 mL of concentrated HCl in a Teflon beaker under continuous stirring. Subsequently, 1.0 g of Ti_3_AlC_2_ powder was gradually added to the solution and the mixture was gently stirred at 38 °C for 48 h to complete the etching process. The suspension was sequentially washed three times with 1 M LiCl solution, three times with 1 M HCl solution, and finally with deionized (DI) water via centrifugation at 5000 rpm until the pH of the supernatant reached approximately 6. The precipitate was re-dispersed in DI water and centrifuged at 3500 rpm for 5 min to obtain a dark green, homogeneous Ti_3_C_2_T_X_ nanosheet suspension.

### Synthesis of PS Templates

PS templates were prepared via an emulsion polymerization method. In a typical procedure, 15 mL of styrene was placed in a separatory funnel and washed with 100 mL of 5 wt% NaOH aqueous solution to remove the stabilizer, followed by three washes with DI water. The purified styrene was transferred into a 500 mL three-neck round-bottom flask containing 100 mL of DI water. Dissolved oxygen was removed by bubbling N_2_ gas for 30 min, and the mixture was maintained at 70 °C with stirring for another 30 min. Then, 0.3 g of K_2_S_2_O_8_ was added, and the reaction was allowed to proceed at 70 °C for 24 h under continuous stirring. The resulting PS spheres were collected by centrifugation at 4000 rpm for 10 min to remove large aggregates.

### Preparation of Aramid Nanofiber (ANF) Dispersion

ANF dispersion was prepared by dissolving 1.0 g of Kevlar® 49 thread and 1.5 g of KOH in 500 mL of DMSO. The suspension was magnetically stirred at room temperature for 1 week to yield a uniform dark red ANF dispersion. The ANF was subsequently dispersed in DI water and ultrasonicated to obtain a homogeneous aqueous dispersion. (The DMSO solvent used for ANF preparation can be recovered and purified for reuse in subsequent processing, enabling sustainable resource utilization. In the laboratory, the used DMSO is collected and stored in dedicated chemical reagent bottles for proper handling and management.)

### Preparation of the Reference Samples

The fabrication of the 3D porous Ni-integrated MXene film (Ni-PMF) involved the following steps. First, a Ti_3_C_2_T_X_ suspension (10 mL, 6 mg mL^−1^) was dispersed in 20 mL of deionized water, followed by the addition of 3 mL of NiCl_2_·6H_2_O aqueous solution (0.1 M). After thorough shaking, 6 mL of NaOH aqueous solution (3 M) containing 0.3 g of NaBH_4_ was introduced into the mixture. The reaction was maintained under continuous shaking for 25 min, after which the resulting solid was collected and washed with deionized water three times to yield Ni–Ti_3_C_2_T_X_ mixed solution. Subsequently, Ni–Ti_3_C_2_T_X_ mixed solution and 1 mL of PS solution were added to 50 mL of ANF solution and stirred uniformly. The mixture was processed into films via vacuum-assisted filtration, followed by lyophilization for 48 h. Finally, the films were annealed at 450 °C for 1 h under an inert atmosphere with a heating rate of 5 °C min⁻^1^ to obtain the Ni-PMF.

To investigate the role of Ni nanoparticles and the porous Ti_3_C_2_T_X_ architecture in the EMW absorption performance of Ni-PMF, comparison samples were prepared as follows: PMF (fabricated without the metallic Ni precursor), Ni-MF (fabricated without PS spheres), and MF (fabricated without both the metallic Ni precursor and PS spheres). In addition, to further demonstrate that the multifunctional performances originate from the synergistic effects of structural and material characteristics, we systematically adjusted the ratio of Ni–Ti_3_C_2_T_X_ mixed solution to PS solution. Under otherwise identical experimental conditions, the concentrations of PS solution were set to 0.5 and 2 mg mL⁻^1^, and the resulting functional films were denoted as Ni-PMF-1 and Ni-PMF-2, respectively.

### Electromagnetic Parameter Measurements

Complex permittivity measurements were recorded from 2–18 GHz using a vector network analyzer (Keysight E5080B). Before measurement, the sample films were prepared by sliced into cylindrical slices of the same dimensions. (The inner diameter and outer diameter are 3.04 and 7.00 mm, respectively. The thickness of the sample film is approximately 1 mm.)

## Results and Discussion

### Fabrication and Characterization of Materials

**Fig. 1:**
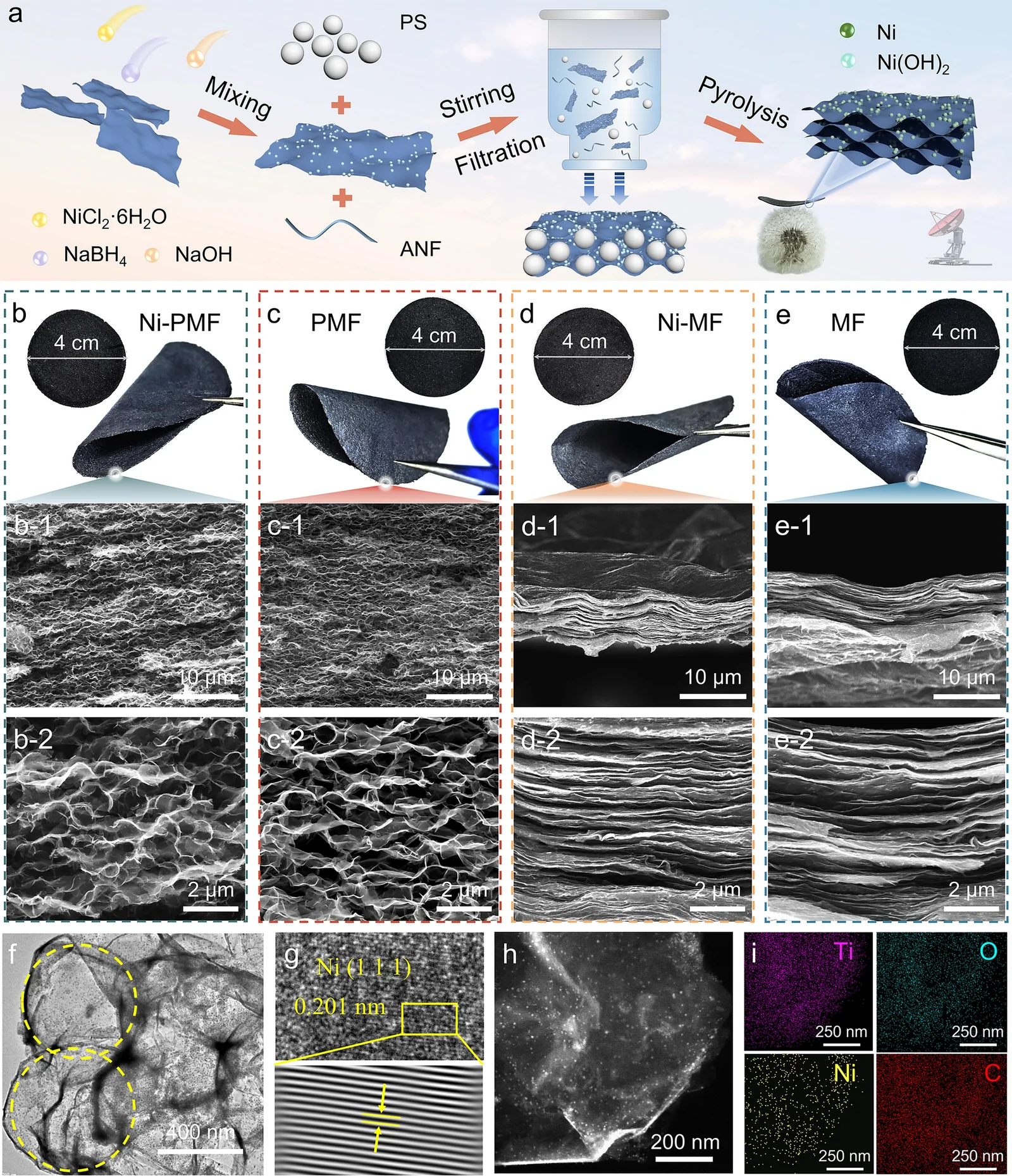
**a** Schematic illustration of the fabrication process of Ni-PMF. Photographs and SEM images of **b** Ni-PMF, **c** PMF, **d** Ni-MF, and **e** MF. **f** TEM image of Ni-PMF. **g** HRTEM image of Ni-PMF. **h, i** EDX elemental mapping images of Ni-PMF

The formation process of the Ni-PMF film is illustrated in Fig. 1a. Initially, few-layered MXene (Ti_3_C_2_T_X_) nanosheets were synthesized by selectively etching the Al layers from Ti_3_AlC_2_ using HCl/LiF solution. Scanning electron microscopy (SEM) images confirm the successful preparation of few-layered MXene, revealing the transformation from the stacked Ti_3_AlC_2_ phase to delaminated nanosheets (Fig. S1). The corresponding X-ray diffraction (XRD) pattern matches well with previously reported MXene structures, further verifying the successful synthesis of MXene nanosheets (Fig. S2). The well-exfoliated MXene nanosheets are rich in surface terminations, such as –OH, –F, and –O, which provide negatively charged sites capable of attracting Ni^2^⁺ ions via electrostatic interactions [28, 29]. Upon hydrolysis and nucleation, these functional groups act as confinement sites for the anchoring and growth of Ni^2+^ species. Ni^2+^ is converted into Ni(OH)_2_ precursors through the reduction reaction with NaBH_4_ and NaOH (Fig. S3). Subsequently, composite films were obtained by blending Ni(OH)_2_-MXene mixture with ANF and PS microspheres in a simple vacuum-assisted filtration process. The PS spheres serve as spacers to introduce hierarchical porosity. During high-temperature annealing at 450 °C, Ni(OH)_2_ is in situ reduced to metallic Ni nanoparticles, and the PS templates are removed. Figure S4 shows the thermogravimetric analysis (TGA) curves of PS spheres, MXene (Ti_3_C_2_T_X_) nanosheets, and the PS/MXene composite. (The PS/MXene composite was prepared by vacuum-assisted filtration of PS microspheres and MXene at a mass ratio of 1:1.) The TGA results indicate that the PS spheres are almost completely decomposed when the temperature reaches 450 °C, whereas MXene (Ti_3_C_2_T_X_) nanosheets exhibit minimal weight loss of only ~ 5 wt% under the same conditions. For the PS/MXene composite with a 1:1 mass ratio, the total weight loss at 450 °C is approximately 50 wt%, confirming that the PS spheres can be effectively removed through thermal treatment at this temperature. In addition, TGA was employed to further investigate the behavior of ANF during the annealing process. As shown in Fig. S4, the TGA curves indicate that ANF begin to decompose between 500 and 600 °C due to the cleavage of amide bonds, which is consistent with previously reported results [30]. Therefore, at 450 °C, ANF does not undergo carbonization. This conclusion is further supported by XRD analysis, as the XRD pattern of the Ni-PMF films after carbonization at 450 °C still exhibits the characteristic peaks of ANF (Fig. S5). The preservation of the ANF structure is beneficial for composite films, as it maintains excellent mechanical strength, flexibility, and dimensional stability. It is worth noting that the fabrication process of the Ni–PMF film is designed to be scalable and environmentally friendly. The DMSO solvent used for ANF preparation can be recovered and reused, while PS microspheres act as a sacrificial template that is completely removed through thermal decomposition without leaving toxic residues. Importantly, the ANF structure is well preserved during annealing, with no carbonization or generation of harmful byproducts. The annealing is performed at 450 °C under a controlled Ar atmosphere, which ensures the formation of a stable conductive network, minimizes energy consumption, and prevents excessive oxidation. These features collectively demonstrate that the proposed method is environmentally compatible and holds potential for large-scale applications. This process results in the formation of a flexible 3D porous MXene framework embedded with uniformly dispersed Ni nanoparticles, generating abundant heterogeneous Ni/MXene interfaces. To elucidate the formation mechanism and the role of each component, a series of control samples were prepared: PMF (fabricated without the metallic Ni precursor), Ni-MF (fabricated without PS spheres), and MF (fabricated without the metallic Ni precursor and PS spheres).

The SEM images of the Ni-PMF film exhibit a well-defined interconnected porous network, as shown in Fig. 1b. High-magnification SEM images confirm that the pore size is approximately 500 nm, consistent with the diameter of the PS spheres used as templates (Fig. S6). This structure originates from the uniform distribution of PS microspheres between MXene layers prior to annealing, forming a sandwich-like architecture. After annealing, the removal of PS templates leads to the retention of highly porous channels, yielding an open porous MXene framework. This design facilitates the penetration of incident EMWs into the interior of the material, extending the propagation path, increasing multiple reflections, and promoting effective energy dissipation. The SEM image further reveals that PMF film retains the open interconnected porous structure similar to that of Ni-PMF (Fig. 1c). In contrast, the Ni-MF and MF films fabricated without PS spheres display tightly packed, parallel layered structures with minimal porosity (Fig. 1d, e). Nitrogen adsorption–desorption measurements further verify the enhanced porosity. The specific surface area of the Ni-PMF film reaches 30.6 m^2^ g^−1^, significantly higher than that of Ni-MF (8.1 m^2^ g^−1^) (Fig. S7). Similarly, the specific surface area of MF without Ni doping increases from 4.0 to 14.1 m^2^ g^−1^ in PMF. This hierarchical porous structure not only contributes to a lightweight design by reducing the material density, but also facilitates the construction of continuous conductive networks for EMW attenuation [31]. Additionally, the high porosity improves impedance matching, and the abundant Ni/MXene heterogeneous interfaces enhance polarization loss [32]. The synergistic combination of these factors provides an effective pathway for optimizing the EMW absorption performance of the Ni-PMF film. Transmission electron microscopy (TEM) image further revealed the porous structure of the Ni-PMF, along with the uniform distribution of small sized Ni nanoparticles on its surface (Fig. 1f). High-resolution TEM (HRTEM) image of the shell region displays clear lattice fringes with a lattice spacing of 0.201 nm, corresponding to the (111) planes of metallic Ni (Fig. 1g). The elemental distribution of Ni-PMF was further examined by scanning transmission electron microscopy (STEM) coupled with energy-dispersive X-ray spectroscopy (EDX). The mapping results confirm the uniform distribution of Ti, Ni, O, and C elements within the MXene matrix (Fig. 1h, i).

**Fig. 2:**
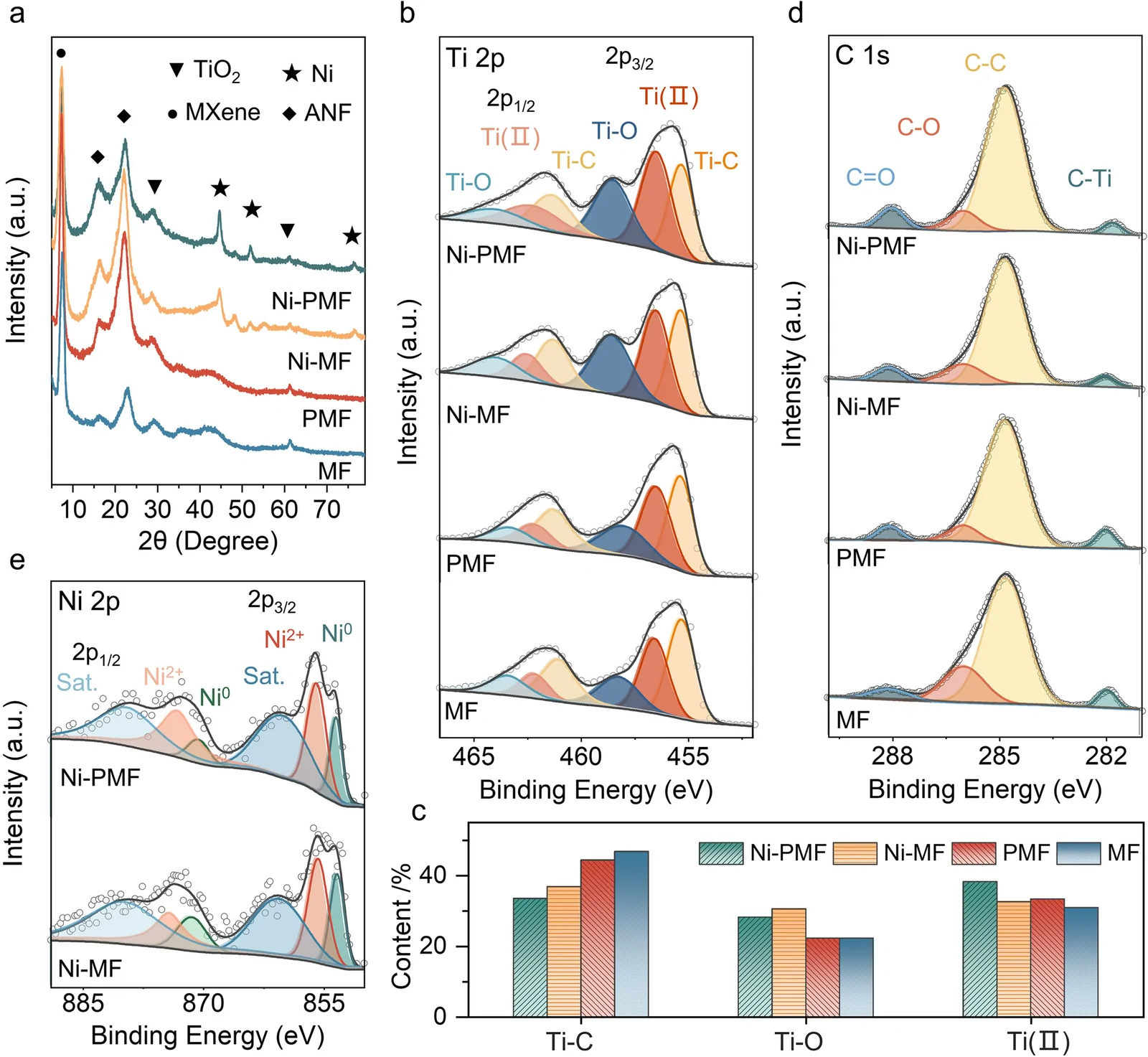
**a** XRD patterns of the samples. **b** High-resolution Ti 2p XPS spectra of the samples. **c** Quantitative analysis of different Ti chemical bond components in the samples. High-resolution **d** C 1*s* and **e** Ni 2*p* XPS spectra of the samples

XRD analysis was performed to confirm the composition and phase purity of the prepared samples. During the annealing process, the in situ reduction of Ni(OH)_2_ to metallic Ni occurs, as evidenced by the appearance of characteristic diffraction peaks at 44.5°, 51.9°, and 76.4°, corresponding to the (111), (200), and (220) planes of face-centered cubic Ni, respectively (JCPDS No. 4–850) (Fig. 2a). These peaks, marked by star symbols in the XRD patterns of Ni-PMF and Ni-MF samples, confirm the successful formation of metallic Ni nanoparticles. All four samples exhibit clear diffraction peaks at approximately 7.5°, corresponding to the (002) planes of MXene [33]. This result demonstrates that the MXene structure is retained after the thermal treatment. Notably, compared with the Ni-MF and MF film samples, the diffraction peak of the MXene (002) plane in Ni-PMF and PMF shifts markedly toward a lower angle, indicating an effective enlargement of the interlayer spacing (Fig. S8). Such an increase is beneficial for enhancing the specific surface area and improving impedance matching [27]. However, additional diffraction peaks at 28.7° and 61.1° are observed, which can be attributed to the formation of TiO_2_ due to partial oxidation of MXene during annealing (JCPDS No. 46–1237). This oxidation is likely caused by residual oxygen-containing functional groups on the MXene surface. Furthermore, diffraction peaks at 16.1° and 22.5° coincide with those of ANF (Fig. S5), indicating that the ANF structure is maintained after calcination at 450 °C [34]. To further investigate the chemical composition and surface states of the Ni-PMF, Ni-MF, PMF, and MF films, X-ray photoelectron spectroscopy (XPS) analysis was conducted. According to the survey XPS spectra, the Ni-PMF and Ni-MF samples contain Ni, Ti, C, and O elements, while Ti, C, and O signals are observed in the XPS spectra of the PMF and MF (Fig. S9). Table S1 summarizes the elemental composition ratios of the samples, as determined from the XPS analysis. The high-resolution Ti 2*p* spectra of the four samples display similar overall features (Fig. 2b) [35]. The relative Ti–O bond content in Ni-PMF and Ni-MF is significantly higher (28.2% and 30.6%, respectively) compared to that in PMF and MF (22.3% for both) (Fig. 2c). This increase can be attributed to the introduction of Ni precursors, where transition-state Ni(OH)_2_ introduces additional oxygen species during the high-temperature annealing at 450 °C synthesis process, which promotes the formation of Ti–O bonds. Correspondingly, Ni-PMF and Ni-MF samples exhibited reduced Ti-C bond content compared to Ni-free counterparts, further corroborating the shift toward Ti–O bonds. It is worth noting that the XPS spectrum of the Ni-PMF film after one month of storage at room temperature (26 °C) and 40% relative humidity shows a Ti–O bond content of 32.1%, which does not exhibit a significant increase compared with the value obtained immediately after preparation (Figs. 2c and S10). This result demonstrates the good environmental stability of the Ni-PMF film. The C 1*s* spectra of the samples reveal the presence of C–Ti, C–C, C–O, and C = O bonds (Fig. 2d) [35, 36]. Notably, the C–Ti bond intensity is reduced in Ni-PMF and Ni-MF compared to PMF and MF, aligning with the observations from the Ti 2*p* spectra and indicating partial oxidation or transformation of Ti–C bonding sites during composite formation. The chemical states of Ni were further examined by high-resolution Ni 2*p* XPS spectra in Ni-PMF and Ni-MF (Fig. 2e). Peaks at approximately 853.4 and 855.7 eV correspond to the Ni^0^ and Ni^2+^ components of the Ni 2*p*_3/2_ orbital, while peaks at 870.8 and 874.1 eV are assigned to the Ni 2*p*_1/2_ orbital [37, 38]. Additionally, satellite peaks located at 859.9 and 880.8 eV indicate the presence of higher valence Ni species, further confirming the coexistence of metallic Ni and residual Ni^2+^ in the system. The magnetic properties of Ni-PMF and Ni-MF were measured by a vibrating sample magnetometer (VSM). Figure S11 displays the magnetic hysteresis loops of Ni-PMF and Ni-MF, which show that both materials exhibit ferromagnetic behavior. The saturation magnetization (*M*_s_) and coercivity (*H*_c_) are 0.60 emu g^−1^ and 9.92 Oe for Ni-PMF, and 0.62 emu g^−1^ and 10.27 Oe for Ni-MF, respectively. The ferromagnetic properties may facilitate their EMW absorption performance [39].

### EMW Absorption Performance

**Fig. 3:**
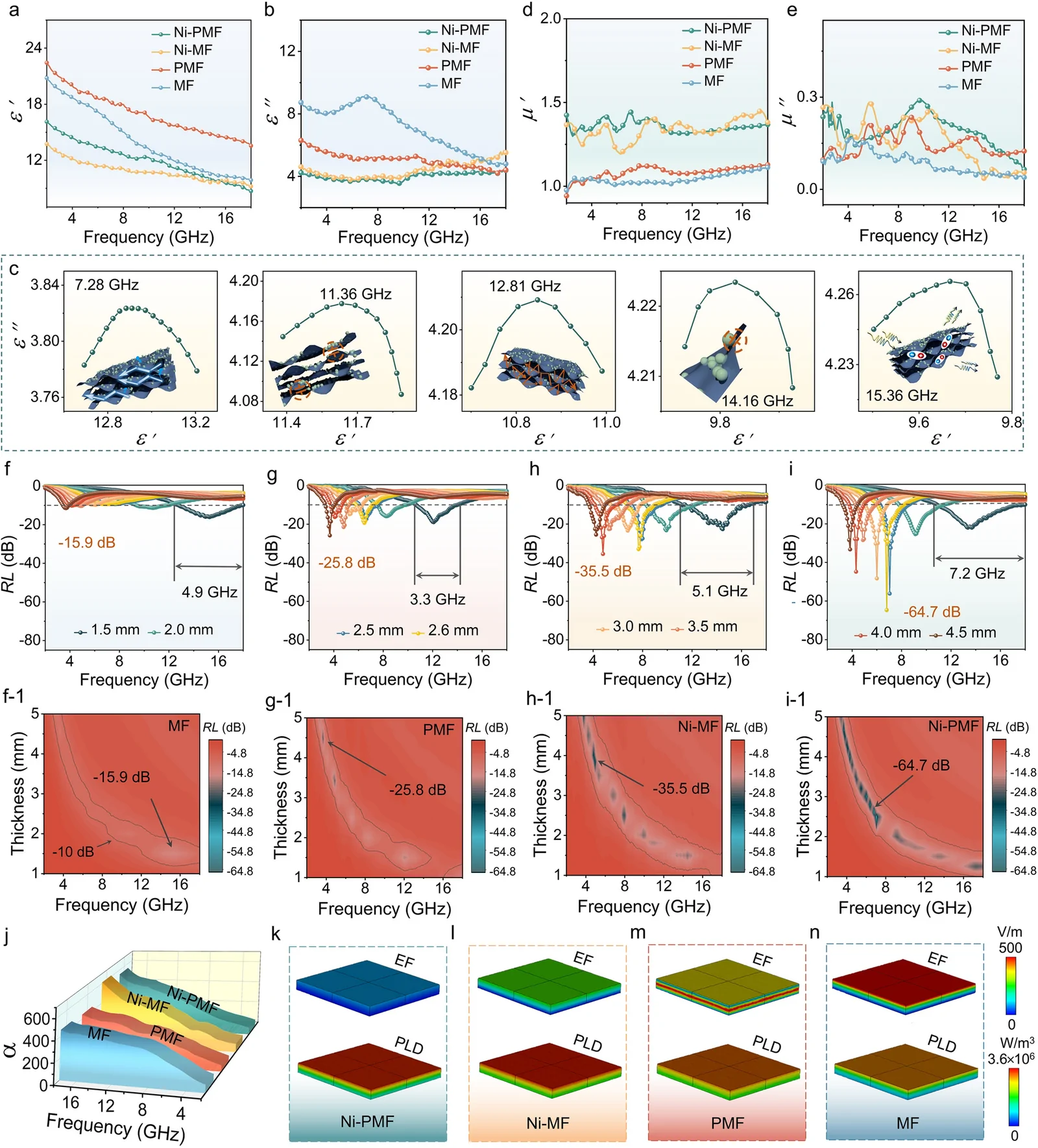
**a, b** Frequency-dependent real (*ε*′) and imaginary (*ε*″) parts of the permittivity for the samples. **c** Cole–Cole plots of the Ni-PMF. **d, e** Frequency-dependent real (*µ*′) and imaginary (*µ*″) parts of the permeability for the samples. *RL* curves of samples: **f** MF, **g** PMF, **h** Ni-MF, and **i** Ni-PMF.** j** Attenuation constant of all the samples. Surface electric field and the power loss densities of **k** Ni-PMF, **l** Ni-MF, **m** PMF and **n** MF

The porous architecture and abundant Ni/MXene heterogeneous interfaces are expected to endow the Ni-PMF films with outstanding EMW absorption capabilities. To further investigate the EMW absorption performances of Ni-PMF and control samples, the complex permittivity (*ε*_r_ = *ε*′ – *jε*″) and magnetic permeability (*µ*_r_ = *µ*′ – *jµ*″) were measured in the frequency range of 2–18 GHz. Generally, the real parts *ε*′ and *µ*′ in these parameters represent the storage capacity of electrical and magnetic energy, respectively, while the imaginary parts *ε*″ and *µ*″ represent the dissipation capacity. As shown in Fig. 3a, the* ε*′ of the samples follow the order of PMF (22.4–13.6) > MF (20.8–9.9) > Ni-PMF (16.1–8.7) > Ni-MF (13.8–9.2). The ε″–* f* curves show that the *ε*″ value variations from 4.2 to 4.4 for the Ni-PMF, from 4.6 to 5.5 for the Ni-MF, from 6.3 to 4.4 for the PMF, and from 8.7 to 4.8 for the MF (Fig. 3b). It can be found that the Ni-incorporated MXene film exhibits a lower dielectric constant compared with the MXene film, which can be attributed to several factors. Firstly, the high dielectric constant of MXene is primarily derived from the polarization of surface functional groups (e.g., –OH, –O) under an external electric field. However, the introduction of Ni may disrupt the uniform surface structure of MXene, reducing the number of effective polarization sites and thereby lowering the overall dielectric constant. Secondly, although Ni nanoparticles can enhance local electrical conductivity, their insufficient dispersion or inability to form a continuous conductive network may limit the overall conductivity of the film. This results in reduced dielectric loss, subsequently influencing the dielectric constant. Thirdly, the dielectric properties of MXene films are closely related to the interlayer interactions. In pure MXene, strong interlayer coupling facilitates high dielectric performance, whereas the incorporation of Ni may weaken these interactions, leading to a more disordered layered structure and diminished dielectric response. It is also worth noting that MXene films with porous architectures (PMF and Ni-PMF) exhibit a higher *ε*' value and a lower *ε*'' value compared with densely stacked MXene films (MF and Ni-MF). This can be explained by the following factors. The porous structure introduces additional interfaces and micro capacitor-like regions, which enhance space charge polarization and thus increase the real part of the dielectric constant. At the same time, the presence of pores disrupts the continuous conductive pathways within the MXene layers, effectively suppressing conduction loss and reducing dielectric loss, reflected in the lower *ε*''. Moreover, the porous morphology facilitates improved impedance matching, allowing better energy storage capability while minimizing energy dissipation [40]. The appearance of relaxation peaks in the *ε*″ curve suggests the occurrence of polarization relaxation, which further analyzed using Cole–Cole plots based on Debye theory. As shown in Figs. 3c and S12, multiple semicircular arcs appear in the Cole–Cole plots, confirming the existence of multiple polarization relaxation processes [41, 42]. This multi-relaxation behavior can be attributed to the heterogeneous interfaces and structural complexity introduced by Ni nanoparticles. Specifically, the interfaces between Ni and MXene generate interfacial polarization, while the inherent dipolar polarization from surface functional groups of MXene also contributes to the dielectric response [43, 44]. Furthermore, defects, vacancies, and localized charge accumulations at the Ni/MXene interfaces introduce additional polarization centers, resulting in multiple relaxations.

In addition to dielectric loss, magnetic loss also plays a critical role in the EMW attenuation process. As shown in Fig. 3d, e, the trends of *μ*′–* f* curves and *μ*″–* f* curves of all the samples are consistent. The introduction of magnetic Ni particles in the MXene films increases the *μ*′ and *μ*″ values over the 2–18 GHz range. These nanoparticles effectively disperse on MXene surfaces, promoting a magnetic coupling network that amplifies magnetic loss. Magnetic loss mechanisms include hysteresis, domain wall resonance, natural resonance, exchange resonance, and eddy current effects. Hysteresis and domain wall resonance, predominant in MHz, are negligible at GHz frequencies [45]. Evaluation of eddy current loss via the coefficient *C*_0_ = *μ*′′(*μ*′)^−2^*f*^*−*1^ reveals significant fluctuations across 2–18 GHz, indicating its minimal role (Fig. S13). Notably, *μ*″–*f* curves exhibit resonance peaks around 6–10 GHz, suggesting magnetic loss primarily derives from natural and exchange resonances. Theoretical calculations using Kittel's equation (Eq. S4) estimate natural resonance frequencies near 3.0 GHz, confirming minimal contribution [46, 47]. Aharoni’s theory (Eq. S5) predicts exchange resonance frequencies around 8.5 GHz, aligning with experimental data [48, 49]. Thus, exchange resonance emerges as the dominant magnetic loss mechanism in Ni-PMF films.

To comprehensively assess the EMW absorption performance of the samples, the reflection loss (*RL*) was calculated based on transmission line theory. The *RL*–*f* curves for different sample thicknesses ranging from 1.0 to 5.0 mm are presented in Fig. 3f–i. The *RL*_min_ of the MF can only get –15.9 dB at 15.0 GHz, which shows poor EMW absorption performance. In comparison, the *RL*_min_ of the PMF sample at 3.7 GHz increased to –25.8 dB with a thickness of 4.5 mm, demonstrating enhanced EMW absorption performance. This indicates that the construction of a 3D pore structure contributes significantly to improving the EMW absorption characteristics. The *RL*_min_ of the Ni-MF can get –35.5 dB at 4.8 GHz, implying that the introduction of metallic Ni contributes to improving the EMW absorption performance. Compared with the control samples, the Ni-PMF sample exhibits superior EMW absorption performance, with the *RL*_min_ of –64.7 dB at 6.9 GHz. In addition, it is clear that the absorption frequency corresponding to *RL*_min_ shifts to lower frequencies as the matching thickness increases. This phenomenon can be explained by the quarter-wavelength cancelation law. As shown in Fig. S14, the *t*_m_ values of the Ni-PMF film generally follow the simulated *λ*/4 curves, suggesting that the phase difference between the incident and reflected waves is 180°, resulting in interference cancelation [50, 51]. Another critical parameter for evaluating EMW absorbers is the EAB_10_, which represents the frequency range over which more than 90% of incident EMW energy is absorbed. As shown in Figs. 3f–i and S15a, the optimal EAB_10_ value of Ni-PMF can reach 7.2 GHz at a thickness of 1.5 mm, which covers the whole Ku-band and is much wider than those of Ni-MF (5.1 GHz at a thickness of 1.5 mm), PMF (3.3 GHz at a thickness of 1.5 mm), and MF (4.9 GHz at a thickness of 1.5 mm), further indicating that the structural regulation and the composition of Ni-MXene affect the EMW absorption characteristics. The Ni-PMF sample demonstrates outstanding EMW absorption capabilities compared to the control samples, as reflected by its significantly lower *RL*_min_ and broader EAB_10_ values (Fig. S15b). This exceptional performance highlights the material potential for meeting the demands of next-generation lightweight and high-frequency electronic communication systems. Compared with the reported MXene-based films (Fig. S16 and Table S2), Ni-PMF delivers a superior combination of wider EAB_10_ and lower *RL*, underlining its promise as an advanced EMW protection material.

For efficient EMW absorption, materials must simultaneously achieve good impedance matching and strong attenuation capability [37, 52, 53]. Impedance matching enables incident EMWs to enter the material with minimal reflection at the surface, while sufficient attenuation ensures that the absorbed waves are effectively dissipated within the material [54]. To quantitatively evaluate the EMW absorption performance, the impedance matching parameter (*Z*) was calculated. When the *Z* value of an absorber approaches 1, the input impedance of the material becomes close to that of free space. This condition minimizes the reflection of incident EMWs at the material surface, allowing more EMWs to penetrate into the absorber and be attenuated within it. To achieve a more comprehensive and quantitative assessment, the impedance matching region defined as 0.8 ≤ *Z* ≤ 1.2 (close to 1) was adopted to evaluate the impedance matching performance [55, 56]. As shown in Fig. S17, the Ni-PMF sample exhibits a broader impedance matching region compared with the control samples, confirming its superior impedance matching capability and enhanced EMW absorption behavior. Notably, even at a reduced thickness of only 1.25 mm, the Ni-PMF still achieves an *RL* value of –27.1 dB, corresponding to over 99% absorption of the incident EMW at this thin configuration (Fig. S18). The EAB_10_ of the Ni-PMF reaches 4 GHz at a thickness of 1.25 mm, further indicating that the film exhibits good EMW absorption performance and maintains effective impedance matching at an ultrathin thickness. In addition to impedance matching, the attenuation constant (*α*) is another critical parameter for characterizing EMW absorption performance. Under the premise of good impedance matching, a higher *α* value indicates a stronger capability of the material to attenuate incident EMW, thereby enhancing the overall EMW absorption efficiency [57, 58]. As presented in Fig. 3j, *α* value can get 62.7–553.4 for Ni-PMF, 75.1–610.5 for Ni-MF, 54.8–443.4 for PMF, and 71.7–460.3 for MF. Based on the above discussion, Ni-PMF shows better impedance matching and appropriate attenuation ability of the incident EMWs than other samples, which endows it with the expected EMW absorption performance. The surface electric field (EF) and power loss density (PLD) distributions of sample films were further simulated to visualize their EM absorption characteristics, as shown in Fig. 3k–n. Surface electric field is commonly utilized to evaluate the impedance matching behavior of EMW absorbers. Regions with reduced red intensity imply that more incident EMW is successfully entering the material, indicating improved impedance matching. Notably, the Ni-PMF film exhibits smaller red regions compared to other reference samples, suggesting its superior capability to minimize reflection and promote EMW penetration [59]. Furthermore, the simulated power loss density distributions provide insight into the attenuation characteristics of the materials. More intense red area corresponds to enhanced EM energy dissipation. Among the compared samples, Ni-PMF displays the pronounced power loss density, highlighting its good EMW attenuation ability. The above results confirm that the excellent EMW absorption performance of the Ni-PMF film stems from its optimized impedance matching and efficient energy dissipation.

**Fig. 4:**
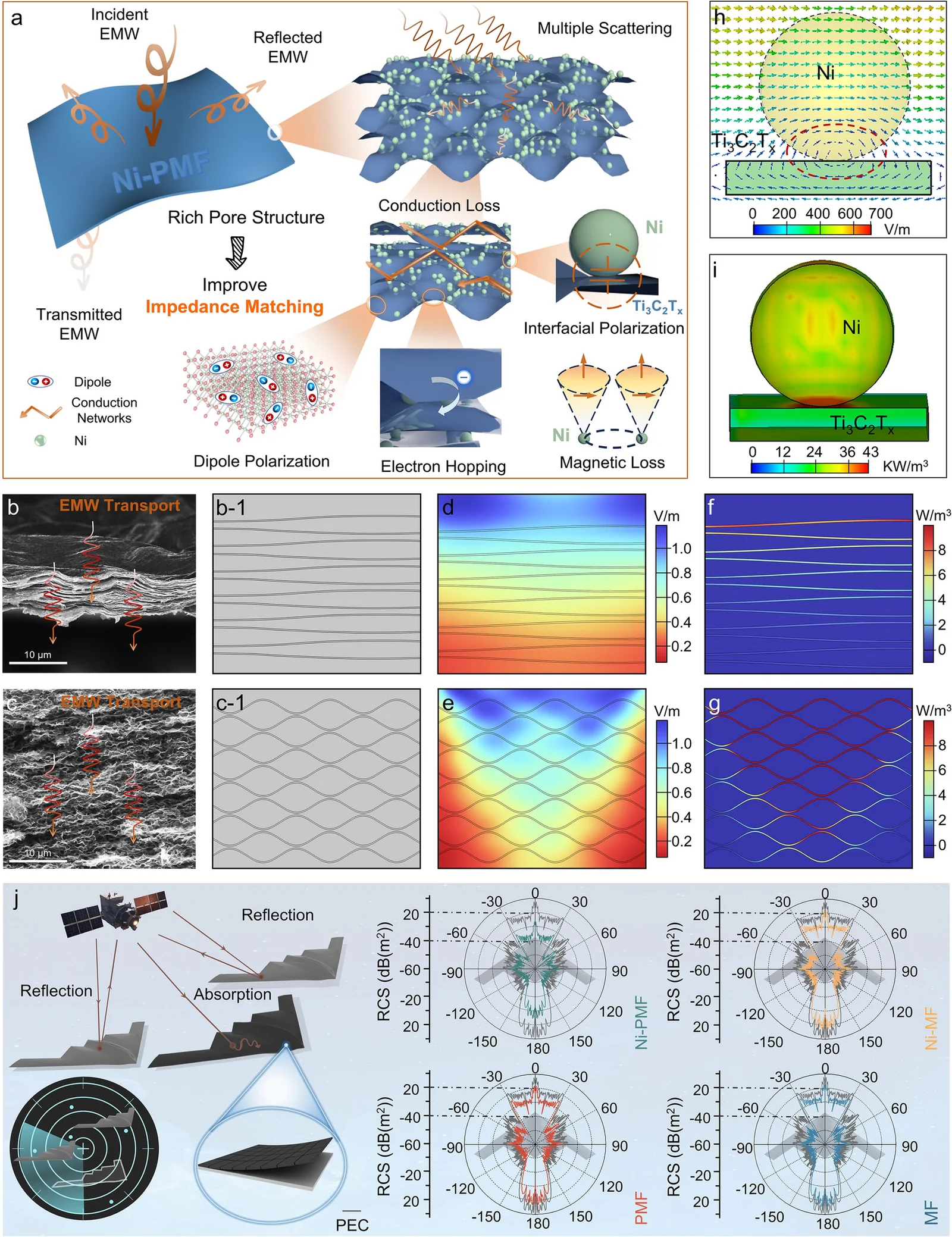
**a** Schematic illustration of the EMW absorption mechanism in the Ni-PMF film. Structural models and schematic diagrams of EMW transmission within the **b** stacked structure and **c** 3D porous structure. Simulated electric field distribution of the **d** stacked structure, and **e** 3D porous structure. Simulated total EM power dissipation inside the **f** stacked structure, and **g** 3D porous structure. Simulation images for **h** electric field and **i** power loss densities between Ni nanoparticle and MXene.** j** Top-view RCS curves of the airplane under VP

A schematic of the EMW absorption mechanism of the Ni-PMF film is illustrated based on the above results (Fig. 4a). The outstanding EMW absorption performance of the Ni-PMF film is attributed to the synergistic effects of its unique structural and compositional features. Firstly, the construction of a 3D porous network significantly improves impedance matching by introducing abundant air gaps and interconnected channels [60]. This design reduces the effective permittivity of the material, minimizes the reflection of incident waves at the air–material interface, and allows more EMW to penetrate into the absorber. To further elucidate the influence of structure on the EMW absorption properties, finite element simulations were conducted to investigate 10 GHz wave propagation within both stacked structure film and 3D porous film (Fig. 4b, c). As the experimentally synthesized material is a 3D porous MXene-based film integrated with metallic Ni, characterized by the synergistic combination of structural porosity and interfacial engineering, direct modeling of the actual structure would greatly increase simulation complexity and computational cost. Therefore, a simplified periodic structure constructed from surfaces was employed to simulate the EM behavior of the 3D porous film based on its SEM images. Similarly, a periodic structure composed of relatively flat planes was used to simulate the EM behavior of the stacked film. While this simplification cannot fully capture the anisotropy of the 3D porous film, it effectively reflects the primary EM interaction mechanisms dominated by the structure, particularly with respect to dielectric loss and power loss density. The simulated distribution of electric fields and total EM power consumption in 3D porous films and stacked structure films under the action of 10 GHz incident EMWs is shown in Fig. 4d-g. In the stacked structure films, the high surface conductivity causes severe impedance mismatch with free space, resulting in the reflection of incident waves and the formation of standing wave effects. This leads to the confinement of the electric field within a narrow region near the interface (Fig. 4d). In contrast, the 3D porous film provides better impedance matching, allowing the incident waves to propagate deeper into the interior of the structure and generating a deep and more uniform electric field distribution (Fig. 4e). This enhances the potential for energy attenuation. The comparison of total power dissipation confirms that, compared to stacked structures, the 3D porous architecture offers longer and more efficient attenuation paths for EM energy (Fig. 4f, g). Therefore, the theoretical simulations further verify that the rational construction of a 3D porous film can substantially enhance the impedance matching characteristics, thereby improving its EMW absorption performance. Simultaneously, the porous structure also facilitates multiple scattering of EMWs, extending the transmission path and increasing energy loss. Secondly, the uniform distribution of Ni nanoparticles within MXene nanosheets generates abundant heterogeneous interfaces, where differences in electrical conductivity, dielectric constant, and Fermi level between the two components lead to asymmetric charge distribution [61]. This difference in charge storage capacity induces the formation of interfacial dipoles [62]. Under an alternating EM field, these dipoles attempt to reorient, but the polarization lags behind the field variation, thereby dissipating EM energy as interfacial polarization loss [63]. Ni nanoparticles are embedded within the porous MXene framework, forming numerous heterogeneous interfaces. In the CST simulation, Ni nanoparticles are represented using a simplified spherical model, whereas MXene nanosheets are modeled as planar sheets. The simulation results further reveal that the electric field distribution at the Ni/MXene interfaces is highly non-uniform, showing clear field gradients and localized enhancement regions (Fig. 4h, i). Such inhomogeneous field distribution reflects charge accumulation and potential differences across the interface, providing direct evidence of interfacial polarization. Moreover, the presence of well-dispersed Ni nanoparticles not only ensures sufficient interfacial regions for polarization but also contributes to magnetic loss through mechanisms such as exchange resonance. The dispersed Ni nanoparticles contribute significantly to both interfacial polarization and magnetic loss, enhancing impedance matching and expanding the EAB_10_. Notably, when pre-oxidized MXene (rich in TiO_2_) was employed as the raw material under identical preparation conditions, the resulting TiO_2_/Ni-PMF films exhibited inferior EMW absorption performance (Fig. S19). This result suggests that TiO_2_ does not play a critical role in the EMW attenuation capability of these composites. In addition, the surface of MXene nanosheets is rich in polar functional groups (–OH, –O, and –F), which act as dipolar centers and generate strong polarization loss under alternating fields. Meanwhile, the Ni-PMF film’s certain degree of electrical conductivity (6.2 S m⁻^1^) facilitates electron hopping and charge transport under alternating EM fields, enhancing conduction loss and thereby promoting the conversion of EM energy into thermal energy [64–66]. Collectively, these synergistic mechanisms—including interfacial polarization, magnetic loss from Ni nanoparticles, dipolar polarization, and conduction loss—endow the Ni-PMF film with outstanding EMW absorption capability.

To further evaluate the practical potential of the prepared materials for EMW stealth applications, especially in military equipment, a full wave simulation was conducted using CST Studio Suite. It should be noted that the CST simulations in this work are performed on an idealized PEC aircraft model, which simplifies real-world effects such as surface curvature, edge diffraction, and multilayer stacking. The simulations are intended to qualitatively demonstrate the relative effectiveness of the prepared film coatings in reducing radar scattering. An aircraft model was employed to simulate the real far-field EM response conditions, in which a 2.6 mm thick sample patch layer was coated on the aircraft surface (Fig. 4j). The simulation framework defines the x-axis positive direction as φ = 0°, the z-axis positive direction as θ = 0°, and the direction of the incident EMW as a function of both θ and φ. In this model, the aircraft is positioned on the x-o-y plane, with the nose pointing along the positive x-axis. Both vertical polarization (VP) and horizontal polarization (HP) conditions were analyzed by extracting the forward view radar cross-section (RCS) profiles and the overall RCS distributions of the aircraft at 6.9 GHz (Figs. 4j and S20–S22). The results show that at 6.9 GHz, the reference model with a perfect electric conductor (PEC) coating exhibits the strongest reflected signal. In comparison, the aircraft model coated with the prepared sample patches exhibits a significantly weakened reflected signal. Among the test samples, the aircraft with Ni-PMF patch layers showed the most significant reduction in RCS compared to the other three materials, thus demonstrating the best radar attenuation performance. The simulation results of these samples are consistent with the EMW loss performance shown in Fig. 3f–i, which further confirms its potential as a promising material for both civil and military stealth coating applications.

### Multifunctional Film and Electrothermal Conversion

**Fig. 5:**
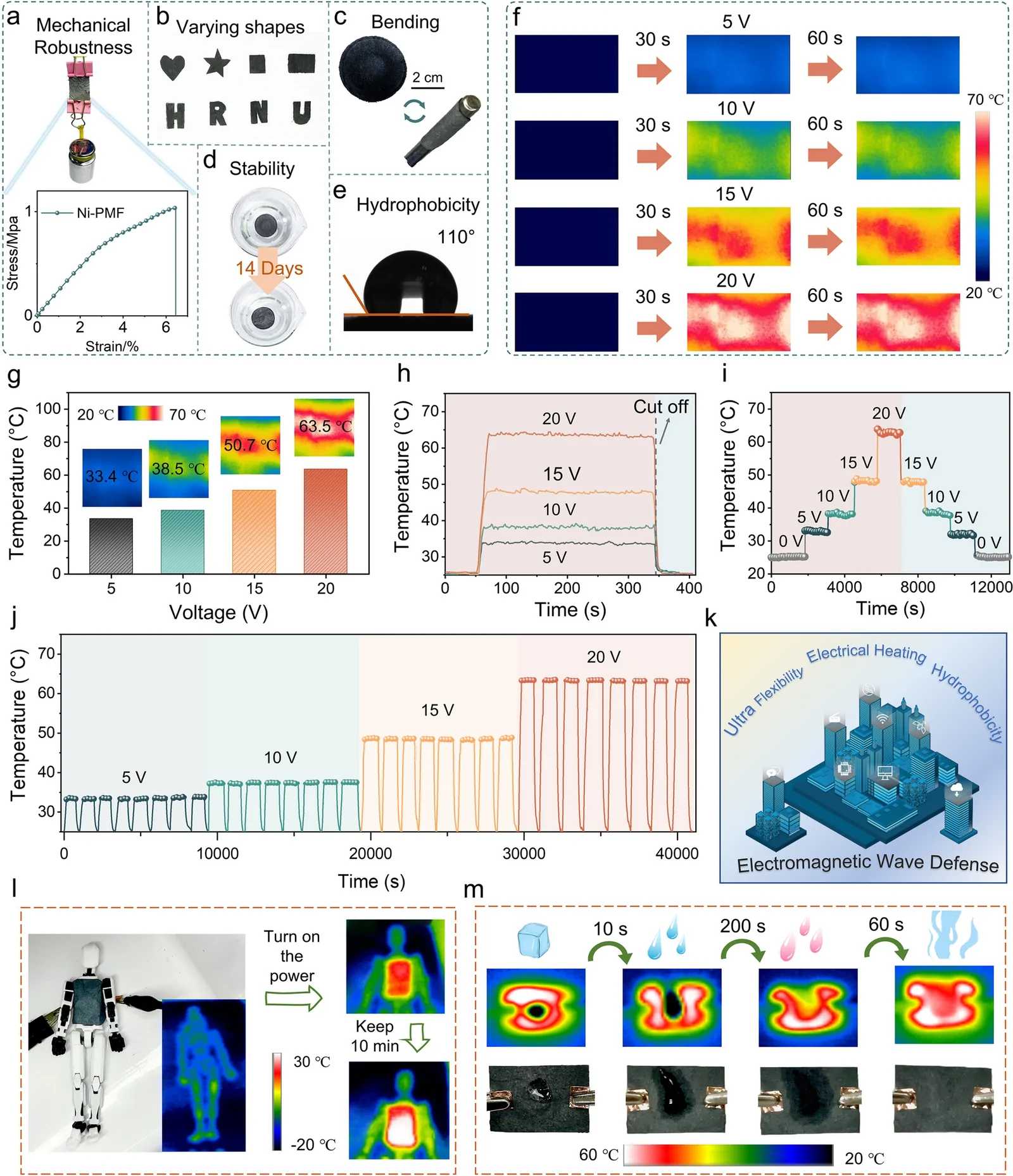
**a** Photograph of Ni-PMF dragging an object 100 g heavier and stress–strain curve of Ni-PMF. Photographs demonstrating the Ni-PMF’s **b** varying shapes, **c** bending flexibility, **d** structural stability, and **e** hydrophobicity. **f** Infrared images, **g** temperature histograms, and **h** temperature profiles of Ni-PMF under applied voltages. **i** Temperature profiles of Ni-PMF during stepwise voltage ramp-up and ramp-down cycles from 0 to 20 V. **j** Heating stability of Ni-PMF during multiple on/off cycles at different voltages. **k** Schematic illustration of Ni-PMF application scenarios. **l** Simulation of Ni-PMF heater performance in cold environment. **m** Photographs and IR images of Ni-PMF during the deicing process at a working voltage of 20 V

In addition to its excellent EMW absorption performance, the unique 3D porous structure of the Ni-PMF film imparts multiple properties that are advantageous for practical applications. Structurally, the film consists of a hierarchical porous framework that integrates a mesoporous skeleton with a multilayered MXene network. This unique architecture not only contributes to weight reduction but also plays a crucial role in regulating the macroscopic mechanical properties of the film [67]. The Ni-PMF film exhibits excellent lightweight characteristics, as demonstrated by its ability to remain stably positioned on delicate dandelion seeds without bending the branches, indicating its ultralight nature (Fig. 1a). The layered porous morphology facilitates uniform stress distribution throughout the structure. As a result, the Ni-PMF film weighing only 30 mg is capable of supporting objects up to 100 g, exceeding 3,000 times its own weight, without rupture or material shedding (Figs. 5a and S23). This remarkable mechanical robustness is further confirmed by tensile tests, which reveal a maximum tensile strength of 1.04 MPa. As shown in Table S3, the Young’s modulus of the composite film is approximately 23.54 MPa, indicating desirable stiffness and load-bearing capability. The tensile stress–strain curve of Ni-PMF at 50% of the maximum strain is shown in Fig. S24. After undergoing 100 cycles of 50% maximum tensile strain, Ni-PMF recovered its original size upon stress release, demonstrating remarkable elasticity without cracking. This further confirms its excellent structural stability. Moreover, bending durability tests revealed that the film retained stable structural integrity even after 100 bending cycles, confirming its outstanding flexibility and reliability (Fig. S25). Moreover, the Ni-PMF film demonstrates excellent designability and processability. As shown in Fig. 5b, it can be easily cut into various shapes without compromising its structural integrity, making it highly adaptable for customized applications. The film also exhibits outstanding mechanical flexibility, it can conform to complex curvatures and recover its original shape after being bent around small diameter rods, showing no visible cracks or damage after deformation (Fig. 5c). In addition to mechanical resilience, the Ni-PMF film displays superior environmental stability. The Ni-PMF film floated stably on water without external support and remained structurally intact after two weeks of immersion, demonstrating excellent resistance to moisture, oxidation, and degradation (Fig. 5d). This is due to the strong covalent bonding within the framework imparts excellent hydrophobicity, with a water contact angle of up to 110°, ensuring reliable performance in high-humidity environments (Fig. 5e) [68, 69]. These features make the Ni-PMF film particularly suitable for applications in harsh or complex service conditions, including humid or corrosive environments.

The Ni-PMF thin film, composed of metallic Ni and MXene nanosheets, exhibits strong potential for a variety of lightweight thermal management applications. Due to its flexible nature, this material is particularly promising for use in areas where efficient heat distribution and flexibility are essential, such as defrosting, defogging, anti-icing, and localized heat therapy [70–72]. To further assess its suitability for these applications, the electrothermal performance of the Ni-PMF films was systematically evaluated. As shown in Fig. 5f, g, the surface temperature of the film can be controlled by adjusting the applied voltage. When voltages of 5, 10, 15, and 20 V were applied, the equilibrium temperatures of film steadily increased to 33.4, 38.5, 50.7, and 63.5 °C, respectively. The Ni-PMF film exhibited rapid heating, reaching steady state temperatures within seconds and maintaining them stably over 300 s without noticeable fluctuations (Fig. 5h), indicating excellent thermal stability. Upon switching off the voltage, the film rapidly cooled down to ambient temperature, highlighting its ultra-fast thermal response and reversible electrothermal conversion behavior. Furthermore, a series of stepwise voltage ramp-up and ramp-down tests from 0 to 20 V confirmed the reliable and controllable temperature adjustment of the Ni-PMF film (Fig. 5i). To assess durability, the film underwent 8 consecutive on/off heating cycles at different voltages over a total duration of 40,000 s (Fig. 5j). The equilibrium temperatures remained consistent throughout the cycles, verifying the excellent stability and reproducibility of its electrothermal conversion performance. In addition to stable heating, the Ni-PMF film also demonstrates excellent flexibility, hydrophobicity, and EMW absorption, making it a promising candidate for multifunctional wearable devices (Fig. 5k). Due to its mechanical compliance, the film can be integrated into wearable garments for applications such as controllable low voltage heat therapy, which can improve blood circulation, relieve muscle pain, or maintain body warmth in cold environments. For example, when a human body model wearing Ni-PMF outerwear was exposed to an extreme cold environment of –20 °C, the surface temperature of the film stabilized at approximately 28 °C under a safe working voltage of 15 V (Fig. 5l). Moreover, the Ni-PMF film exhibits outstanding deicing performance. When a piece of ice was placed on the film surface, it completely melted within 10 s under 20 V (Fig. 5m). Importantly, even after the ice transitioned to liquid, the electrothermal function of film remained unaffected. The residual water was evaporated through continuous heating for 200 s, and the film surface became completely dry after an additional 60 s, demonstrating robust heating stability even in humid conditions. In summary, the Ni-PMF film combines flexible EMW absorption with rapid and stable electrothermal conversion, positioning it as an ideal candidate for advanced wearable applications. These include heating garments for extreme environments such as deep space exploration or Antarctic expeditions, as well as multifunctional equipment requiring both anti-icing and EM interference protection [73, 74].

### Flexible Strain Sensor Performance

**Fig. 6:**
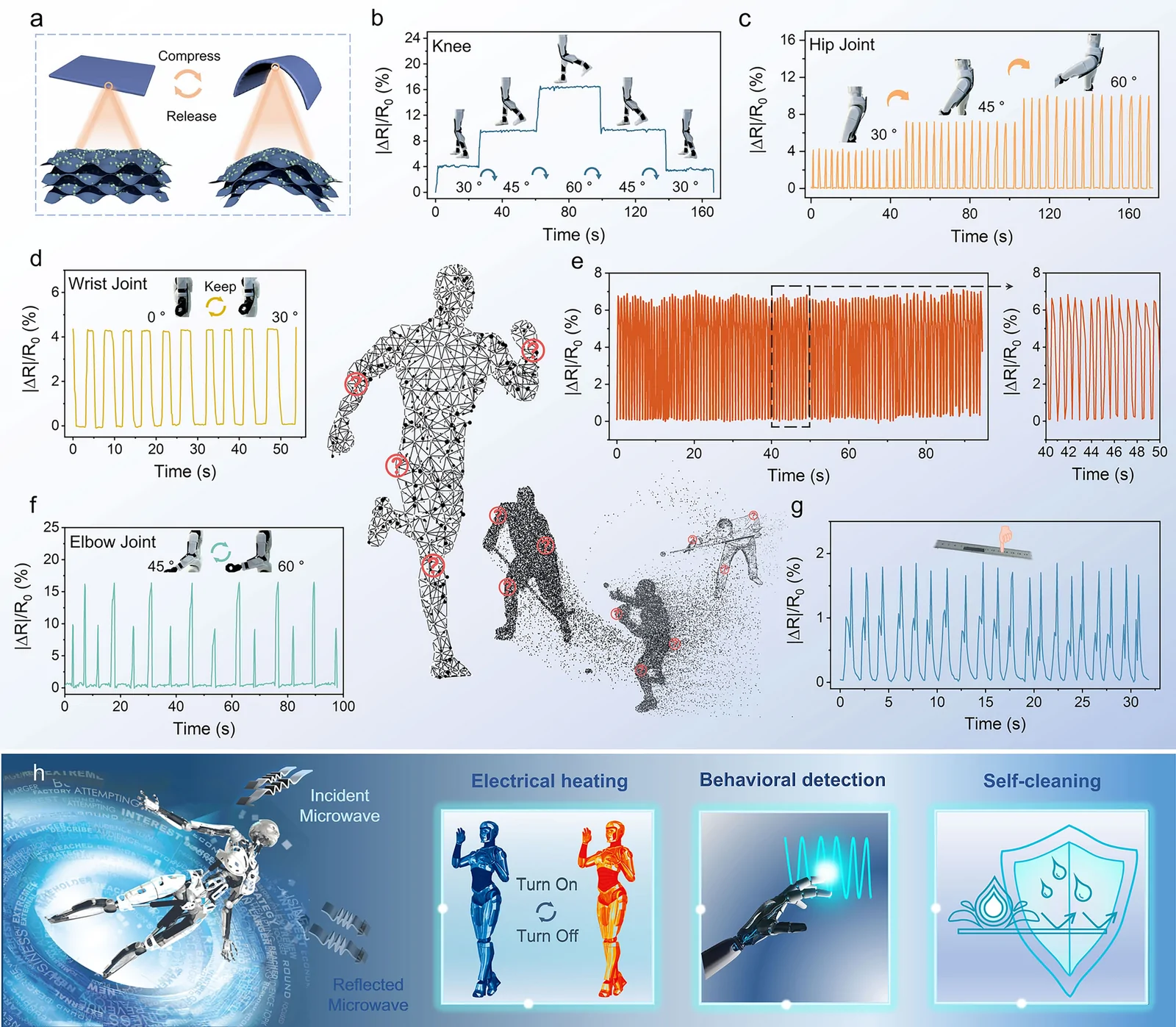
**a** Bending-induced stretch and compression modes of the Ni-PMF. Recorded relative resistance change of the strain for **b** knee, **c** hip joint, **d** wrist joint, **e** repeatability, **f** elbow joint, and **g** ruler. **h** Schematic overview of the multifunctional capabilities of Ni-PMF, including radar stealth, electrical heating, behavioral detection, and self-cleaning

In addition to its outstanding EMW absorption and thermal management performance, the Ni-PMF film also functions as a flexible, durable, and wearable sensor capable of real-time, continuous, and non-invasive monitoring of human motion [75]. This multifunctional capability arises from the high electrical conductivity, excellent mechanical flexibility, and robust structural integrity of the film. To demonstrate its sensing application, electrodes were fabricated at both ends of the Ni-PMF film using conductive silver adhesive and subsequently connected to external circuits with a voltage of 1 V via silver wires. It is noteworthy that at 1 V, the surface temperature of the Ni-PMF film remained the same as the ambient temperature, indicating that the applied voltage did not generate sufficient Joule heating to raise the film temperature (Fig. S26). Therefore, under this condition, the cross-talk between the electrothermal signal and the strain signal can be disregarded. This simple yet effective design enables the Ni-PMF film to operate as a reliable motion detection sensor, expanding its potential for wearable electronics and smart healthcare systems. The Ni-PMF film features a 3D architecture composed of open, interconnected hollow microspheres. During bending, this porous network undergoes structural deformation, resulting in simultaneous stretching and compression on opposite sides of the film (Fig. 6a) [76]. These mechanical deformations alter the conductive pathways within the structure, leading to corresponding changes in electrical resistance [36]. The degree of resistance variation (|*R*–*R*_0_|/*R*_0_%, *R*_0_ is the resistance of Ni-PMF in its unbent state, and *R* is the resistance of Ni-PMF at a certain moment) increases with the bending angle, allowing the sensor to distinguish between different magnitudes of motion based on the relative resistance change [77]. When attached to various joints, including knee, hip, wrist, and, elbows the Ni-PMF sensor successfully captured real-time signals corresponding to limb movements (Fig. 6b–f). For instance, when the knee or wrist was flexed to a predetermined angle and then returned periodically, the sensor output exhibited cyclic and repeatable resistance changes (Fig. 6b, d). A consistent signal pattern was maintained for each identical bending angle, indicating excellent reproducibility and stability in the sensing response (Fig. 6c, f). Moreover, the sensor demonstrated outstanding sensitivity by accurately detecting rapid and repeated bending motions without signal degradation (bending angle is approximately 45 degrees) (Fig. 6e). Beyond joint motion monitoring, the Ni-PMF sensor also detected subtle vibrations, such as those generated by a vibrating ruler (Fig. 6g). By attaching the film to the midpoint of the ruler, the sensor could sensitively capture the mechanical vibrations through strain induced resistance changes, demonstrating its potential for precise vibration detection. This high monitoring sensitivity, coupled with stable and repeatable response characteristics, suggests that the Ni-PMF sensor holds great promise for applications in wearable healthcare devices, robotic prosthetics, tremor disorder management, and rehabilitation monitoring [75, 78]. Thus, Ni-PMF exhibits a combination of advanced functionalities, including high performance EMW absorption, electrical heating, human behavioral detection, and self-cleaning properties (Fig. 6h). The multifunctional performance of Ni-PMF films can be attributed to the synergistic contributions of their structural features and intrinsic material properties. For EMW absorption, Ni nanoparticles provide magnetic loss through exchange resonance, while the MXene matrix contributes multiple dielectric polarization mechanisms, and the hierarchical porous/conductive architecture ensures impedance matching and multiple scattering. For electrothermal conversion, the conductive MXene framework enables efficient electron transport and rapid Joule heating, further enhanced by the well-dispersed Ni nanoparticles. For flexible strain sensing, the layered and deformable structure allows variations in inter-sheet and Ni–MXene contacts under external strain, resulting in resistance changes with high sensitivity. These coupled electronic, magnetic, and mechanical effects endow the Ni-PMF film with excellent multifunctional capabilities. To further demonstrate that these multifunctional performances arise from the synergistic effects of structural and material characteristics, we systematically tuned the ratio of Ni–Ti_3_C_2_T_X_ mixed solution to PS solution. Since this ratio directly determines the film’s composition and microstructure, it plays a critical role in shaping EMW absorption, electrothermal conversion, and flexible strain-sensing behaviors. A series of Ni-PMF films with varying Ni–Ti_3_C_2_T_X_-to-PS ratios were prepared and characterized. SEM observations revealed distinct structural features (Fig. S27): Ni-PMF-1, with fewer PS spheres, exhibited a relatively compact and less porous architecture, providing continuous conductive pathways but limited space for multiple scattering. In contrast, Ni-PMF-2, containing a higher PS content, displayed a highly porous structure with larger and interconnected voids, which favored multiple scattering but partially weakened the conductive network due to excessive porosity. These structural differences highlight the critical role of PS content in balancing porosity, conductivity, and structural stability. The impact of these structural variations on multifunctional performance was evident. EMW absorption measurements indicate that, compared with Ni-PMF, Ni-PMF-1, and Ni-PMF-2 films exhibit poorer *RL* and a thinner EAB_10_ (Fig. S28). This can be attributed to the fact that both insufficient and excessive porosity lead to impedance mismatch, which compromises EMW attenuation. In electrothermal conversion, Ni-PMF-1 reached 41.6 °C under a 10 V applied voltage, whereas Ni-PMF-2 reached 37.7 °C (Fig. S29), likely due to its overly porous framework reducing electrical conductivity and affecting the temperature. For flexible strain sensing, the extent of resistance change indicates that Ni-PMF-1 has poor strain sensitivity due to its low porosity. However, Ni-PMF-2, with a higher porosity, shows enhanced strain sensitivity but has poor stability under repeated bending cycles (Fig. S30). Among these films, Ni-PMF achieved the best overall multifunctional performance, effectively balancing porosity, conductive network integrity, and mechanical stability. Collectively, these results demonstrate that precise control of the composition and microstructure of Ni-PMF films is essential for optimizing their EMW absorption, electrothermal conversion, and flexible strain-sensing performances. It is also noteworthy that the Ni-PMF film maintains stable EMW absorption performance during multifunctional applications. For example, after undergoing a 20 V voltage deicing thermal management process, the Ni-PMF film retains its EMW absorption stability (Fig. S31a). Furthermore, after approximately two weeks of structural stability testing and 80 strain-sensing cycles under a bending angle of about 45°, the film still exhibits good EMW absorption performance (Fig. S31b, c). These results confirm its robustness and reliability for long-term practical applications. With such stable and durable characteristics, the Ni-PMF film demonstrates great potential for integration into next-generation intelligent systems. Its unique structural design enables RCS reduction for stealth applications, while excellent electrical conductivity ensures efficient thermal management against extreme temperatures and fluctuations. At the same time, superior flexibility allows real-time monitoring of subtle limb motions and complex human activities, and the hydrophobic surface guarantees stable operation in humid or contaminated environments [79]. Overall, the multifunctional properties of Ni-PMF not only fulfill the diverse requirements of advanced intelligent electronics but also greatly expand the application prospects of EMW functional materials in cutting-edge technological fields.

## Conclusions

In summary, this work presents a novel 3D porous Ni-integrated MXene film (3D Ni-PMF) with multifunctional capabilities for next-generation EM protection and intelligent sensing applications. By constructing a hierarchical porous architecture using sacrificial templates and incorporating Ni nanoparticles, the Ni-PMF overcomes the limitations of conventional MXene films, including nanosheet restacking and impedance mismatch. The synergistic effect of structural and compositional features enables enhanced multiple scattering, dielectric loss, and magnetic loss, resulting in superior EMW absorption performance with a *RL*_min_ of –64.7 dB and an ultrabroad EAB_10_ of 7.2 GHz, achieving full Ku-band coverage. Furthermore, the film exhibits excellent electrothermal conversion performance and flexible strain-sensing behavior, demonstrating real-time response capabilities. This multifunctional material system not only provides an effective solution for high performance EMW absorption but also offers promising potential for stealth technologies, flexible electronics, and wearable sensing devices, paving the way for next-generation smart electronic systems.

## Supplementary Information

Below is the link to the electronic supplementary material.

Supplementary file1 (DOCX 11588 KB)
