# Supplementary material for: Multifunctional Three-Dimensional Porous MXene-Based Film with Superior Electromagnetic Wave Absorption and Flexible Electronics Performance
Source: Nanomicro Lett. 2026 Jan 5;18:184. doi: 10.1007/s40820-025-02034-2 (PMC12765780; doi:10.1007/s40820-025-02034-2)
Supplement: Supplementary file 1 — Supplementary file1 (DOCX 11588 KB) [file 40820_2025_2034_MOESM1_ESM.docx]

Supporting Information for

**Multifunctional Three-Dimensional Porous MXene-Based Film with Superior Electromagnetic Wave Absorption and Flexible Electronics Performance**

Li Chang^1^, Xinci Zhang^1,^ *, Tingting Liu^1^, Benyi Li^1^, Ying Ji^1^, Gongming Sun^1^, Ziming Wang^1^, Xitian Zhang^1^, Maosheng Cao^2,^ *, Lin Li^1,^ *

^1^Key Laboratory for Photonic and Electronic Bandgap Materials, Ministry of Education, School of Physics & Electronic Engineering, Harbin Normal University, Harbin 150025, P. R. China

^2^School of Materials Science and Engineering, Beijing Institute of Technology, Beijing 100081, P. R. China

*Corresponding authors: [lil@hrbnu.edu.cn](mailto:lil@hrbnu.edu.cn) (Lin Li); [caomaosheng@bit.edu.cn](mailto:caomaosheng@bit.edu.cn) (Maosheng Cao); [zhangxinci@hrbnu.edu.cn](mailto:zhangxinci@hrbnu.edu.cn) (Xinci Zhang)

**S1 Material characterization**

Scanning electron microscopy (SEM, Hitachi SU-70, 15 kV) and transmission electron microscopy (TEM, FEI-Tecnai F20) were performed to characterize the morphology and microstructure. The crystal structure was characterized by X-ray diffraction (XRD) on Rigaku RU-D/max 2500 using Cu Kα radiation (40 kV, 30 mA). Raman spectroscopy measurements were recorded on a Horiba HR-800 confocal Raman spectrometer with an Ar^+^ laser (488 nm). X-ray photoelectron spectroscopy (XPS) characterization was conducted on a ESCALAB 250Xi XPS microprobe with Al Ka radiation. N_2_ adsorption-desorption experiments were carried out on a Micromeritics ASAP 2010 micropore size analyzer at the liquid N_2_ temperature (77 K). Before the measurement, the samples were degassed for 10 h at 150 C. The specific surface area was calculated using the Brunauer–Emmett–Teller (BET) method. The compression modulus of sample was evaluated using a universal testing machine (INSTRON). The water contact angles of the samples were measured using a PT-705C contact angle meter (Dong Guan Precise Test Equipment Co., China). The magnetic properties of the samples were investigated using a CFMS-14T vibrating sample magnetometer (VSM, Cryogenic Co., UK) at room temperature. Thermalgravimetric analysis (TGA) was performed by TG/DTA 6300 system. Young’s modulus, maximum tensile strength, fatigue life, and bending cycles were determined by the universal machine (INSTRON 5982). The conductivities of the samples were determined by the X3 Hall Effect Test System (Semishare International Limited). The electrothermal performanc of the film was tested by thermal infrared imagers (FL-TR T600 series). The water contact angle of the film was measured by the OCA 100.

**S2 Finite element simulations**

To investigate the influence of 3D porous structures and stacked structures on electromagnetic wave absorption performance, we used Comsol Multiphysics 6.2 to simulate the electric field and electromagnetic power dissipation in 3D porous structures and stacked structures under the influence of 10 GHz electromagnetic waves. The electromagnetic simulation model has a width of 1500 μm, a height of 1000 μm, pore size of 10 μm, and a pore spacing of 10 μm. The upper boundary was set with a scattering boundary condition for incident electromagnetic waves at a wavelength of 10 GHz, while the remaining boundaries were set with scattering boundary conditions with no incident electromagnetic waves. The mesh was divided into free triangular meshes, with a total of 22,880 mesh elements and an average element mass of 0.8009. The electromagnetic wave frequency-domain physical field was used for the solution, with the equations as follows:

$\nabla\times\mu_{r}^{-1}(\nabla\times E)-k_{0}^{2}(\in_{r}-\frac{j\sigma}{\omega\epsilon_{0}})E=0$ (S1)

$E(x,y,z)=\tilde{E}(x,y)e^{-ik_{z}z}$ (S2)

**S3 CST simulation**

To assess the practical application of these four samples, further simulations based on far-field response were performed using CST Studio Suite 2022. Four models were simulated. The simulation frequency was set to 9 GHz for all models. For the surface current density and power loss density simulations, the sample films were modeled as a rectangular prism with a length and width of 10 mm and a thickness of 2 mm. The direction of the electromagnetic wave was reversed from the direction of the z-axis. Simulations were performed using a unit cell structure template with a metamaterial full-structure workflow. The relative permittivity and permeability values of each component were input based on experimental measurements from vector network analyzer data. Frequency-dependent parameters were used to better reflect real material responses. The simulation results, including surface current distributions and power loss density maps, were qualitatively compared with experimental results (e.g., reflection loss spectra and impedance matching behavior) to validate the loss mechanism interpretation.

To analyze the sample’s behavior under realistic far-field conditions, simulations were performed using the CST Studio Suite 2022 software package. The electrical boundary was defined as an open boundary condition, and the excitation frequency was carefully selected to be 6.9 GHz. Considering practical application requirements, a simplified aircraft model was employed for far-field radar cross-section (RCS) simulations. Detailed calculations were conducted to determine the interaction between the incident electromagnetic wave and the absorption coating on the aircraft model at various angles and under different motion states. In the simulation framework, the positive x-axis corresponds to φ = 0°, the positive z-axis corresponds to θ = 0°, and the incident electromagnetic wave direction is expressed as a function of both θ and φ. In this model, the aircraft is positioned on the x–o–y plane, with the nose pointing along the positive x-axis. Both vertical polarization (VP) and horizontal polarization (HP) conditions were analyzed by extracting the forward-view RCS profiles and the overall RCS distributions of the aircraft at 6.9 GHz.

**S4 Electromagnetic parameter measurements：**

The Cole−Cole equation can be inferred as:

$\left( \varepsilon^{'}-\frac{\varepsilon_{s}+\varepsilon_{\infty}}{2} \right)^{2}+\left( \varepsilon^{''} \right)^{2}=\left( \frac{\varepsilon_{s}-\varepsilon_{\infty}}{2} \right)^{2}$ (S3)

The natural resonance frequency (*f*_r_) has been calculated by Kittel equation which is expressed as following equation:

$2\pi f_{r}=\gamma H_{0}=\gamma\frac{4\left| K_{1} \right|}{3\mu_{0}M_{s}}$ (S4)

where *γ* is the gyromagnetic ratio, *H*_0_ is the magnetic crystal anisotropy field, *K*_1_ is the magnetic crystal anisotropy constant, *µ*_0_ is the permeability of vacuum and *M*_s_ is the saturation magnetization.

The exchange resonance *f*_r_ can be calculated by Aharoni’s theory which can be described as:

$\omega=\gamma\left( \frac{2A\mu_{kn}^{2}}{D^{2}M_{s}}+H_{c} \right)$ (S5)

where *A* is the exchange constant, *µ_kn_* is the roots of the differential spherical Bessel functions, *D* is the size of metal particles and *H*_c_ is the coercivity in the magnetostatic field.

The reflection loss (*RL*) is determined in the context of the transmission line theory: as follows:

$RL(dB)=20\log\left| \frac{Z_{\mathrm{in}}-Z_{0}}{Z_{\mathrm{in}}+Z_{0}} \right|$ (S6)

The matching impedance (*Z*) of an absorber need to be the following the equation:

$Z=\frac{Z_{\mathrm{in}}}{Z_{0}}=\sqrt{|\frac{\mu_{r}}{\varepsilon_{r}}|}\tan h\left[ j\left( \frac{2\pi fd}{c} \right)\sqrt{\mu_{r}\varepsilon_{r}} \right]$ (S7)

where *Z*_in_ is the normalized input impedance of the absorber, *Z*_0_ is the free space impedance; *ε*_r_ and *μ*_r_ are the permittivity and permeability of the absorber; *f* is the microwave frequency, *c* is the light velocity, and *d* is the thickness of the absorber.

The attenuation constant (α) is defined by the expression below:

$\alpha=\frac{\sqrt{2}\pi f}{c}\sqrt{\left( \mu''\varepsilon''-\mu'\varepsilon' \right)+\sqrt{\left( \mu''\varepsilon''-\mu'\varepsilon' \right)^{2}+\left( \mu'\varepsilon''+\mu''\varepsilon' \right)^{2}}}$ (S8)

The quarter‐wavelength law expressed by the following formula:

$t_{m}=\frac{n\lambda}{4}=\frac{nc}{4f_{m}({|\mu_{r}||\varepsilon_{r}|)}^{\frac{1}{2}})} (n=1,2,3\ldots)$ (S9)

where the *t*_m_ represents the thickness of the absorber, and the *f*_m_ stands for the peak frequency of the *RL* value.

**S5 Supplementary Figures and Tables**


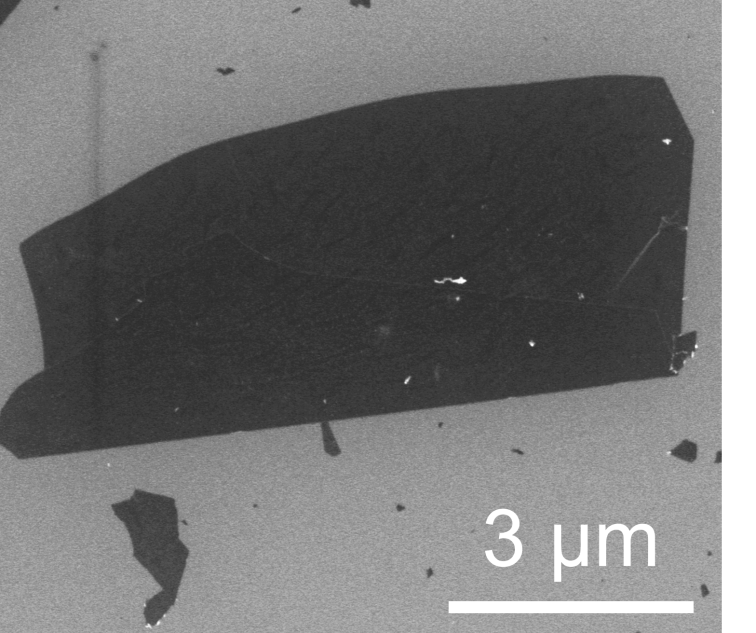


**Fig. S1** SEM image of MXene nanosheet.


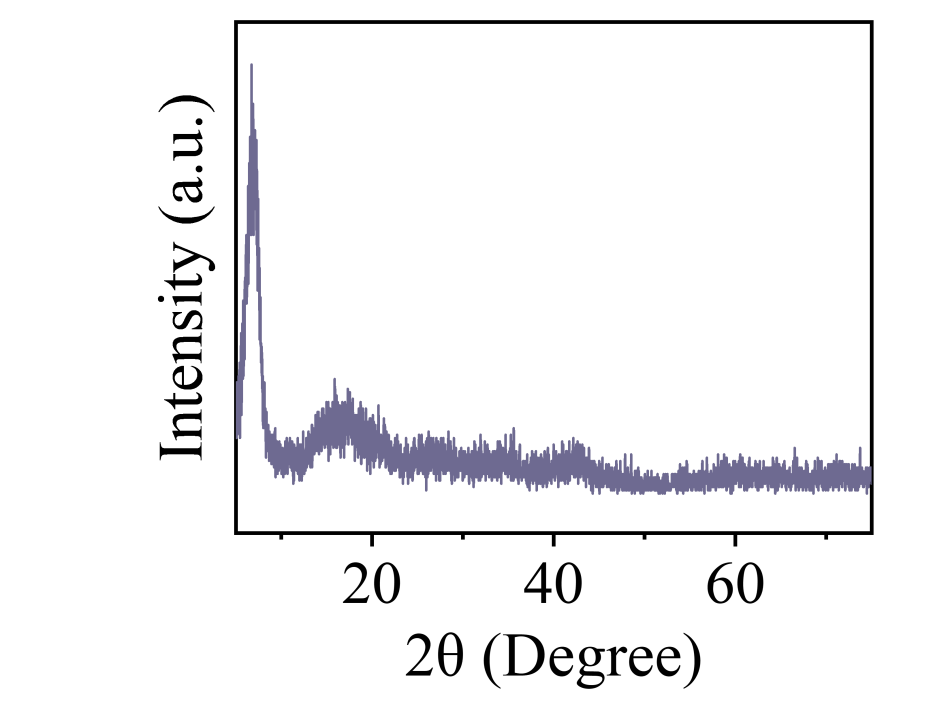


**Fig. S2** XRD pattern of MXene nanosheets


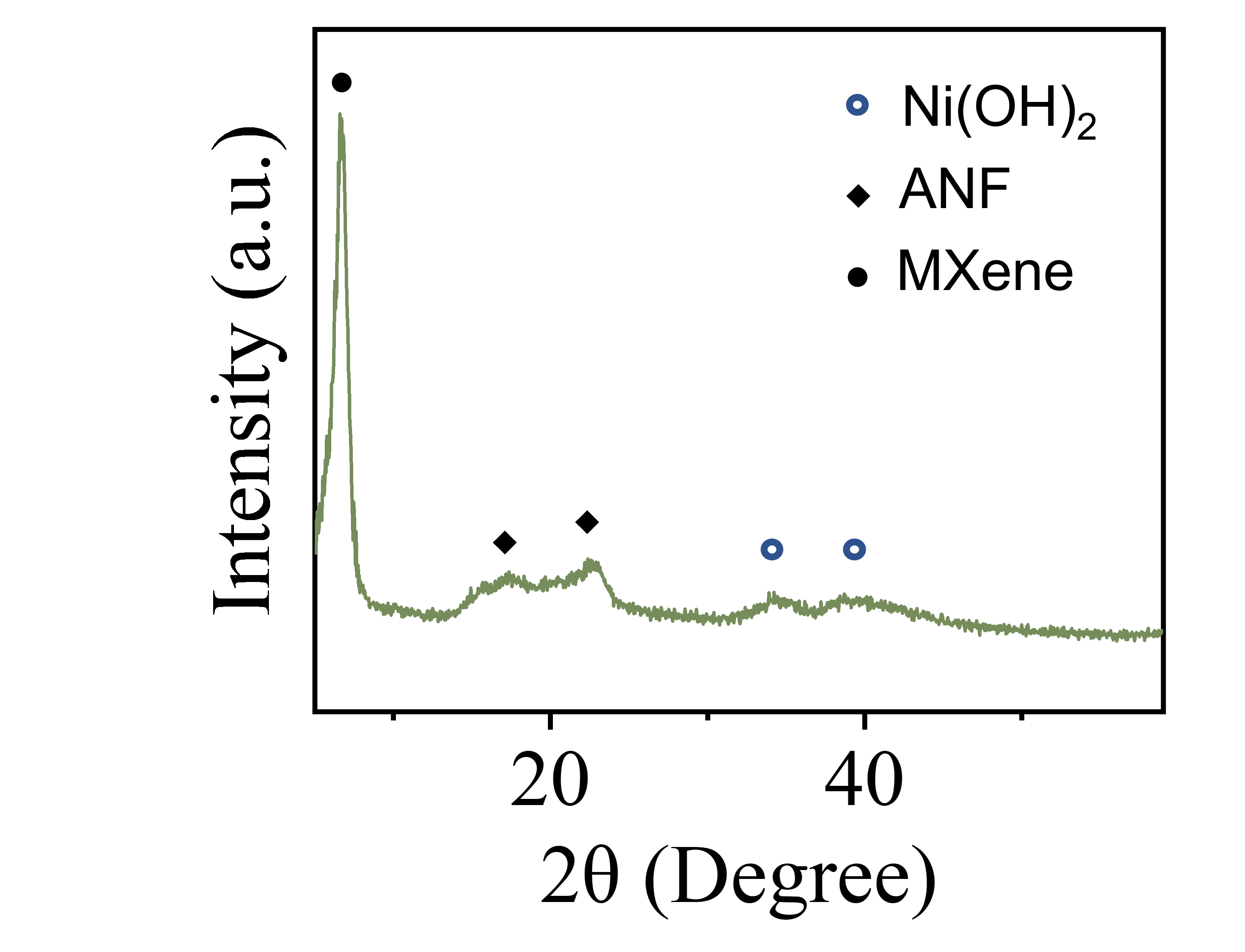


**Fig. S3** XRD pattern of Ni–MXene precursor


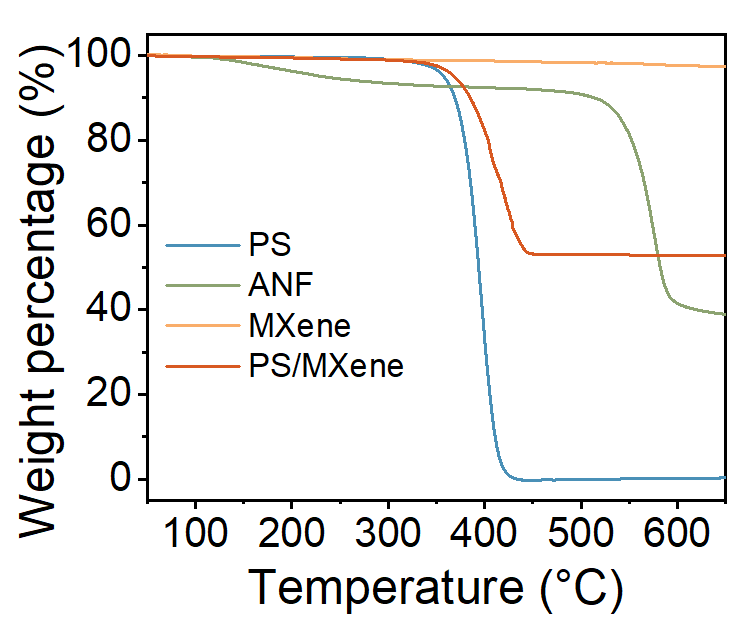


**Fig. S4** TGA curves of the ANF, PS spheres, MXene (Ti₃C₂Tₓ) nanosheets, and the PS/MXene composite


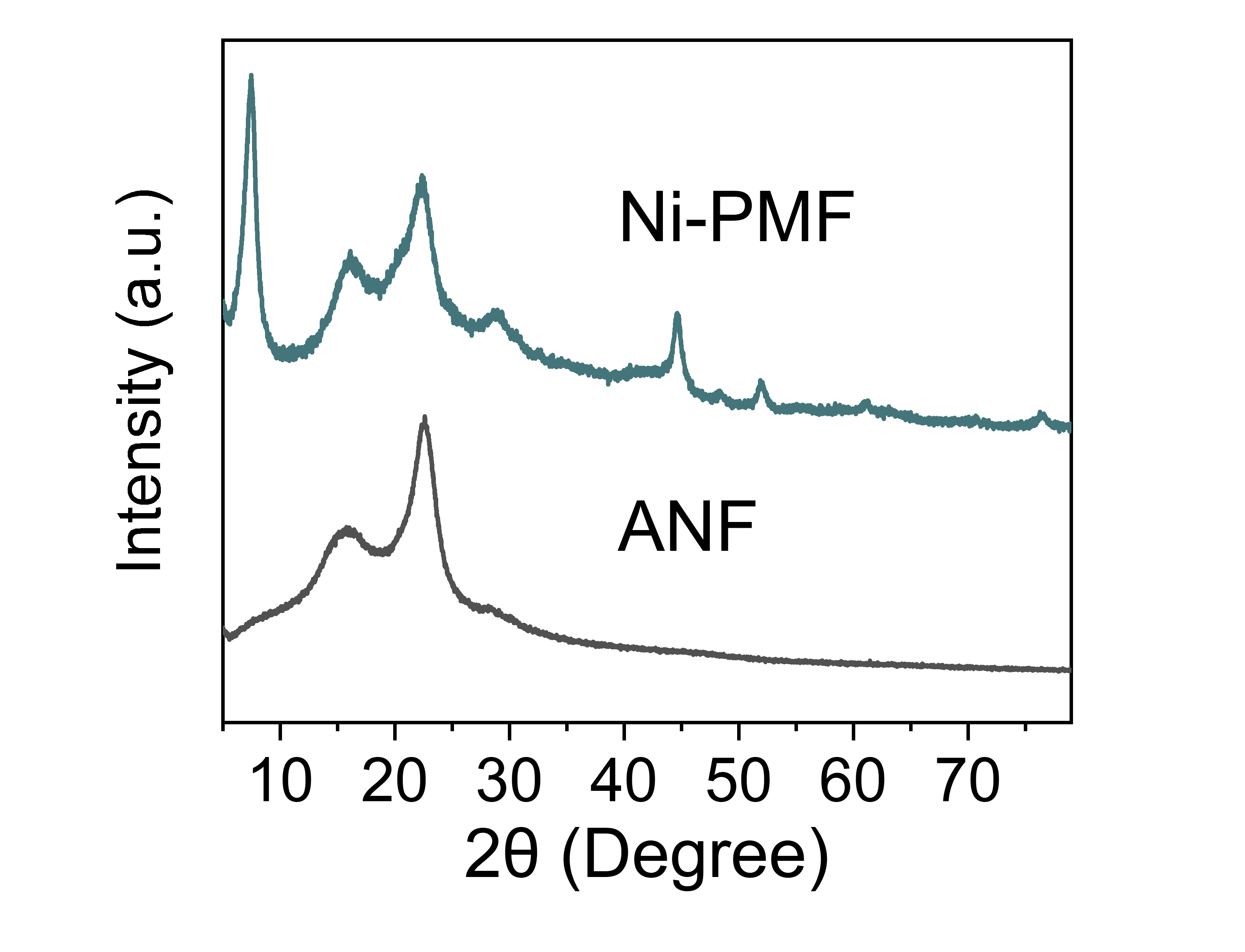


**Fig. S5** XRD patterns of ANF sample and Ni-PMF


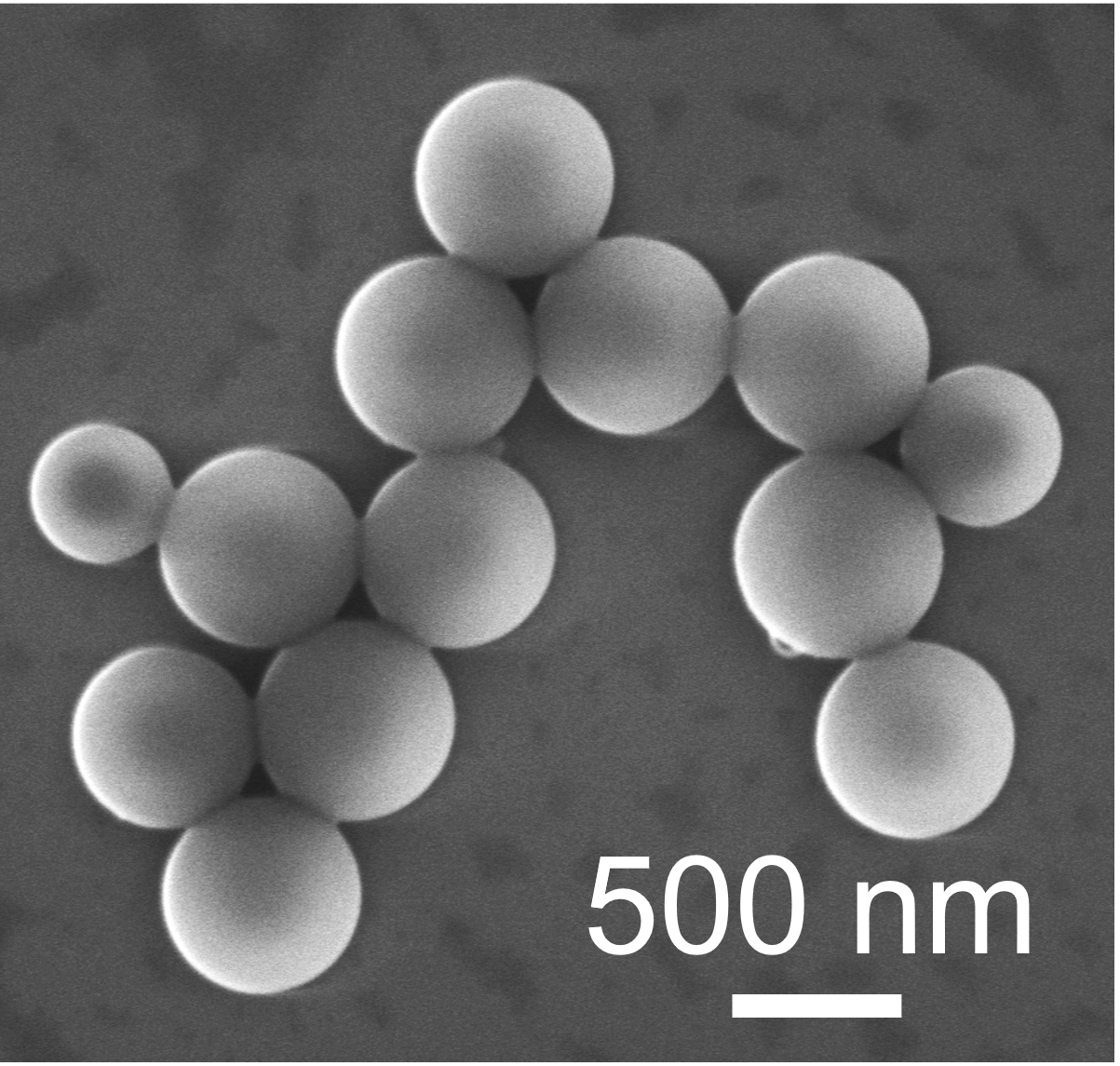


**Fig. S6** SEM image of PS spheres


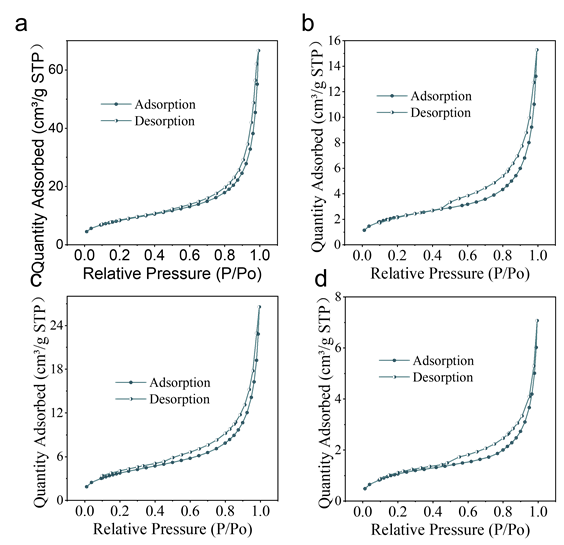


**Fig. S7** N_2_ adsorption–desorption isotherms of **a** Ni-PMF, **b** Ni-MF, **c** PMF, and **d** MF

**
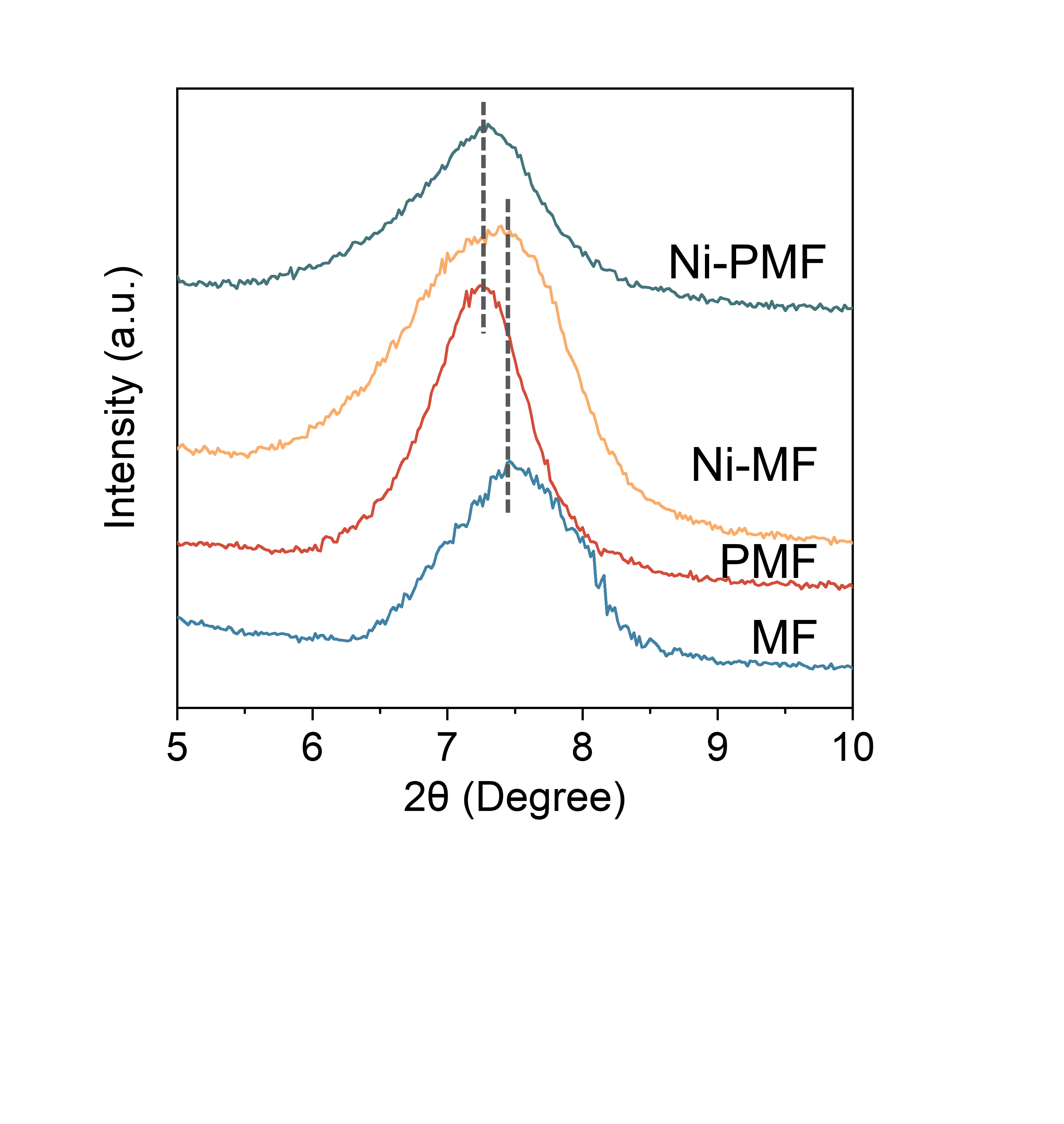
**

**Fig. S8** Magnified XRD patterns of samples

**
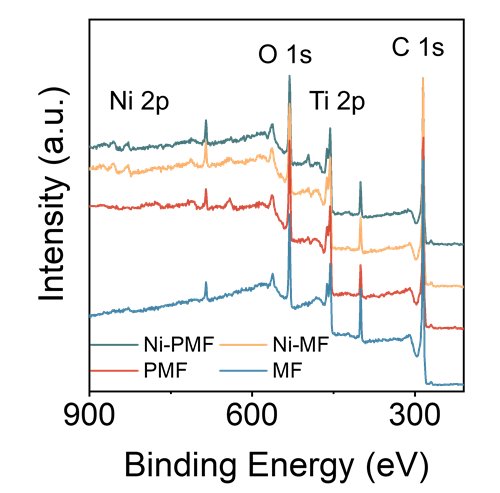
**

**Fig. S9** XPS survey spectra of samples


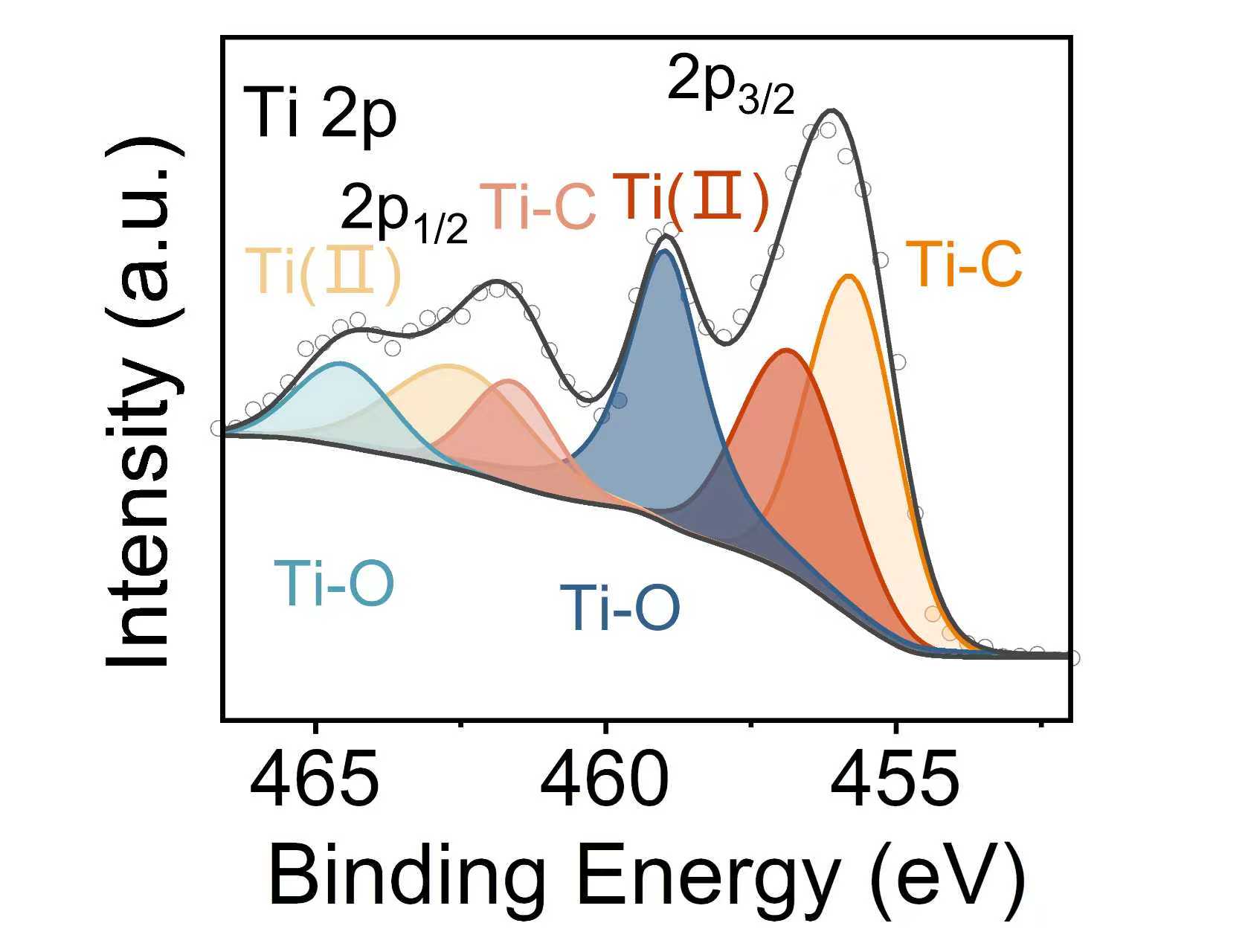


**Fig. S10** High-resolution Ti 2p XPS spectra of Ni-PMF film after storage at room temperature (26 °C) and 40% relative humidity for one month


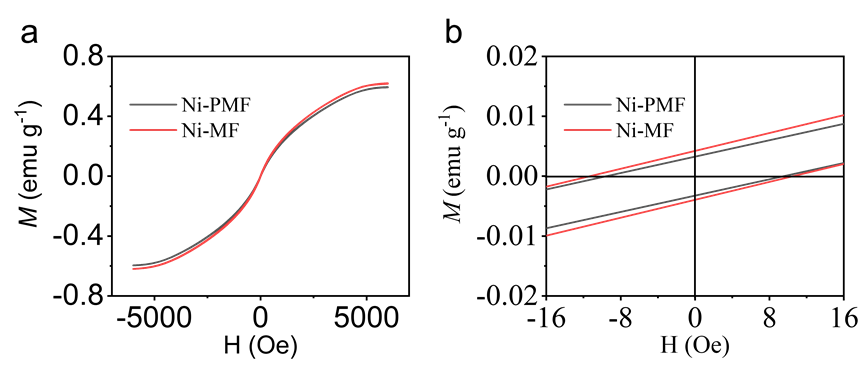


**Fig. S11** Hysteresis loop of Ni-PMF and Ni-MF at room temperature


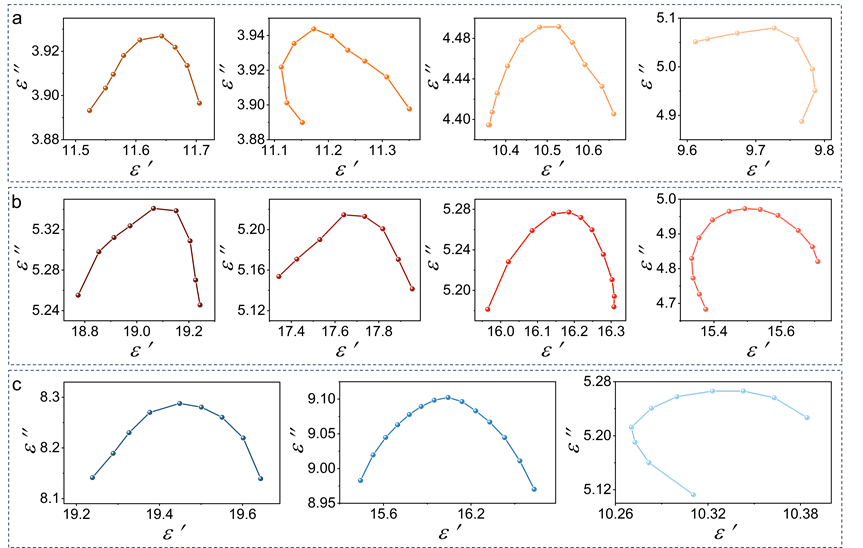


**Fig. S12** Cole–Cole plots of **a** Ni-MF, **b** PMF, and **c** MF


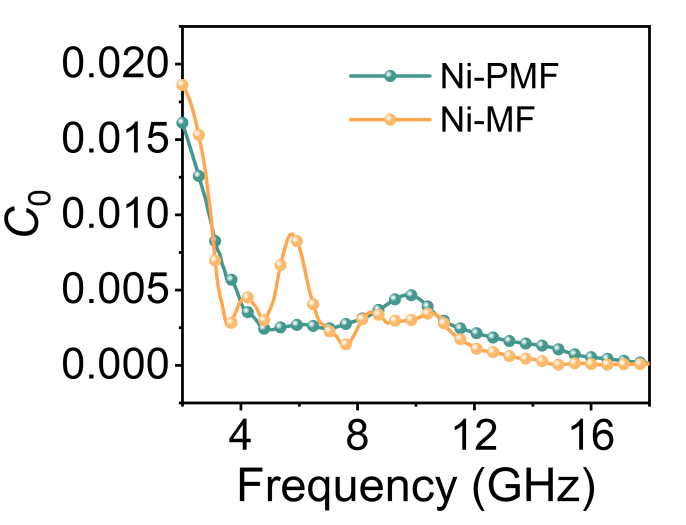


**Fig. S13** The *C*_0_ -*f* curves of Ni-PMF, and Ni-MF


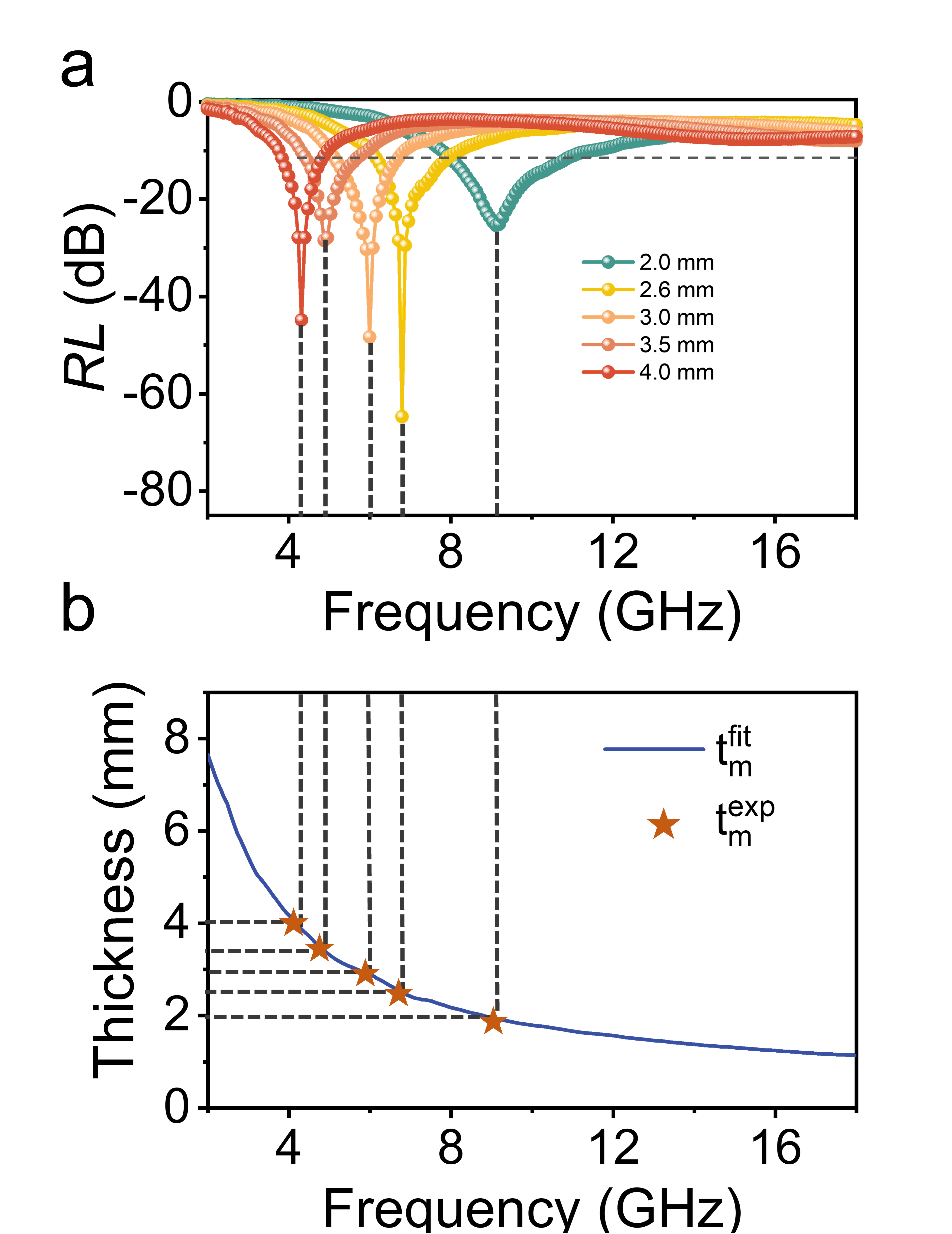


**Fig. S14** **a** *RL* curves of Ni-PMF. **b** Frequency-dependent *RL* and simulations of the matching thickness (*t*_m_) vs. corresponding frequency under the λ/4 model for Ni-PMF, with pentagram representing the experimental *t*_m_


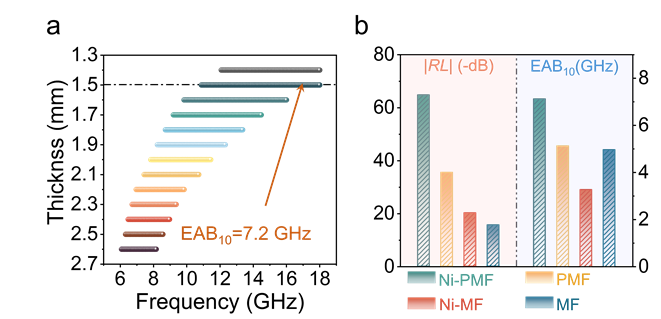


**Fig. S15 a** The maximum EAB_10_ for Ni-PMF at the range from 1.3 to 2.7 mm. **b** The comparison of maximum |*RL*| and EAB_10_ for samples


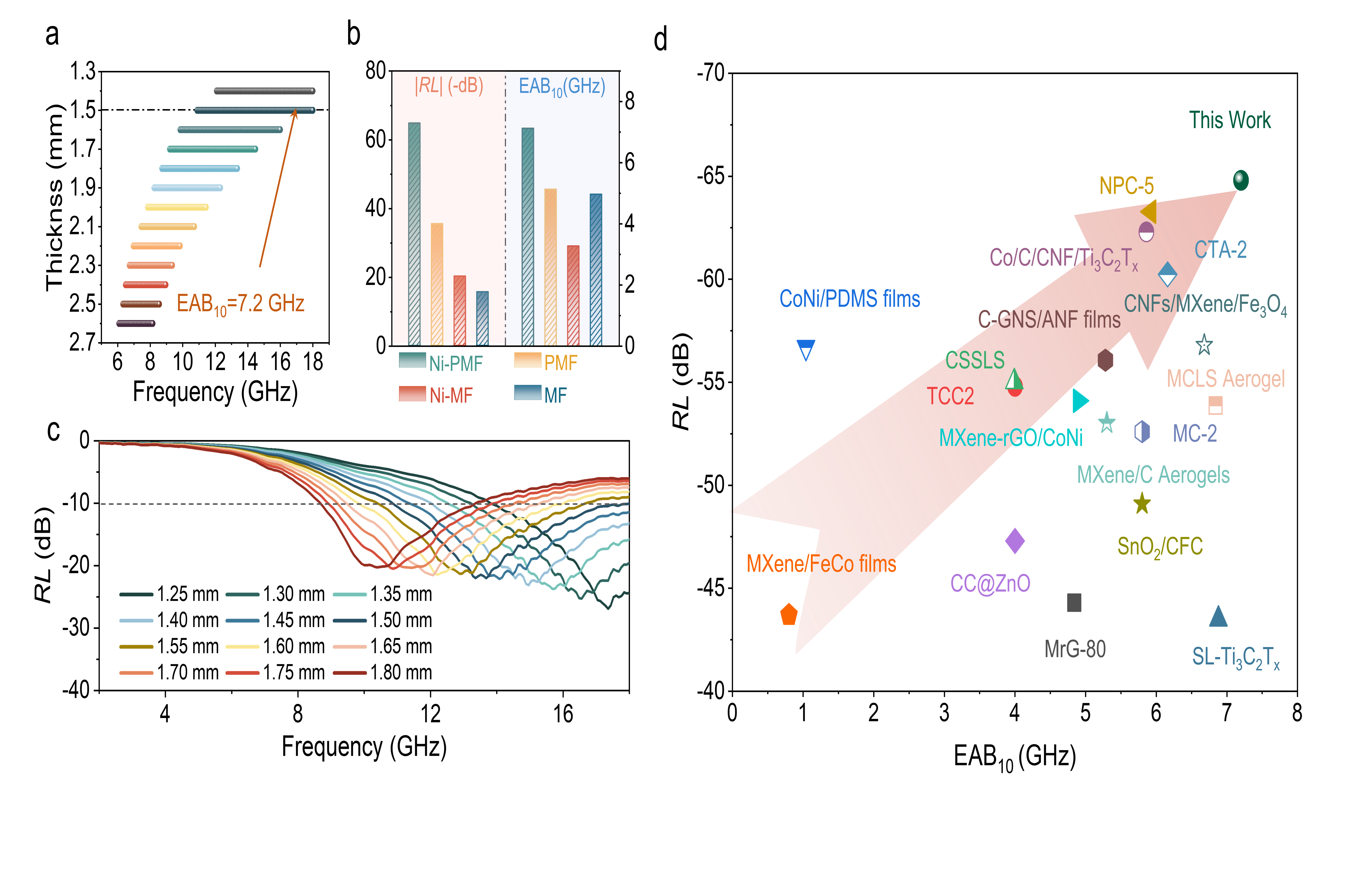


**Fig. S16** Comparison of the *RL*_min_ and EAB_10_ with other reported EMW absorbers


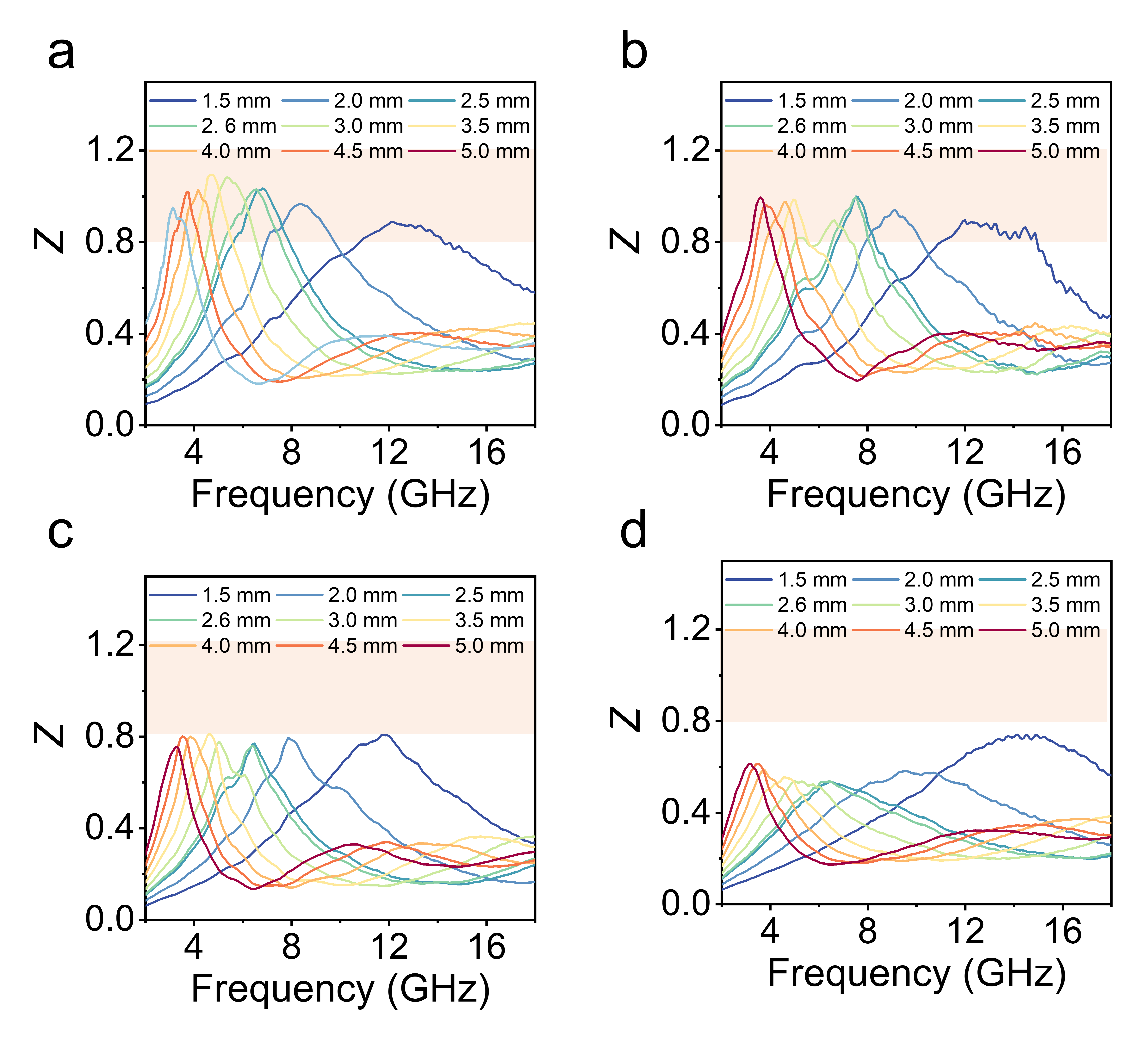


**Fig. S17** Impedance matching images of **a** Ni-PMF, **b** Ni-MF, **c** PMF, and **d** MF


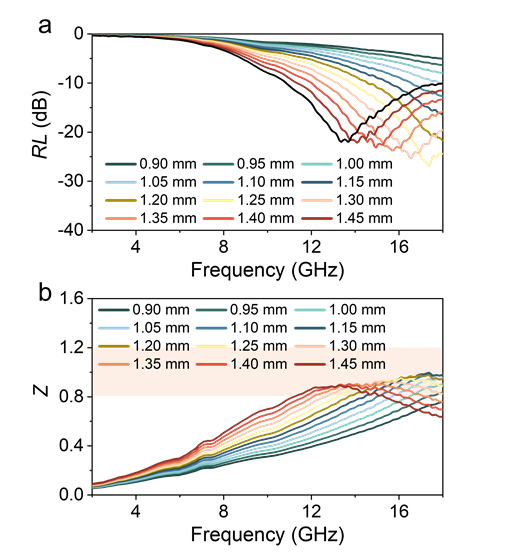


**Fig. S18 a** 2D *RL* curves and **b** impedance matching image of Ni-PMF at the thickness less than 1.5 mm


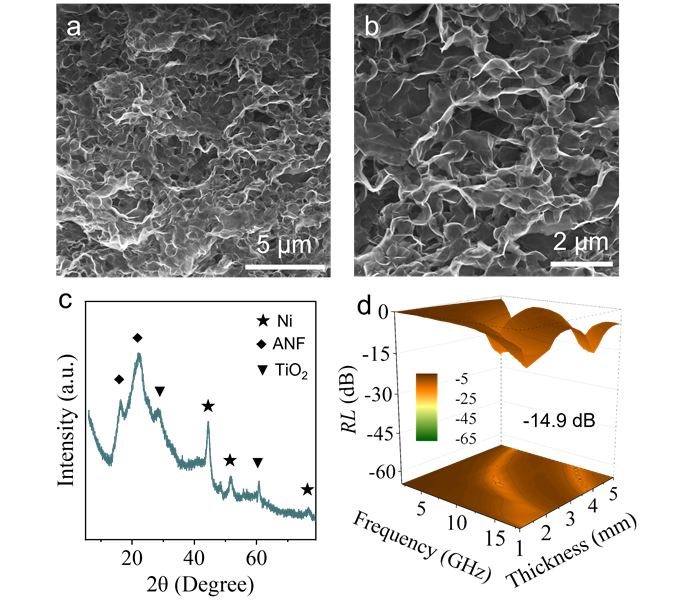


**Fig. S19 a, b** SEM images, **c** XRD pattern, and **d** *RL* curve of oxidized Ni-PMF sample synthesized with pre-oxidized MXene (rich in TiO₂) as the precursor under identical preparation conditions. SEM images reveal that it has a similar 3D porous structure to Ni-PMF. Furthermore, its XRD does not show the characteristic peaks of MXene, but rather those of TiO_2_, indicating that it is completely oxidized


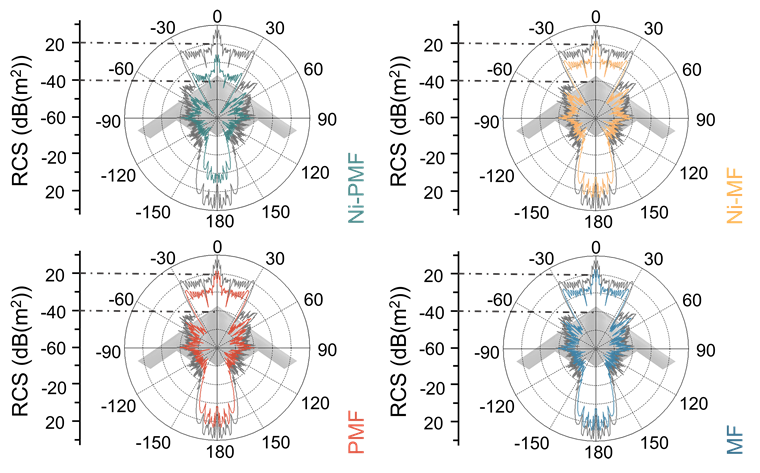


**Fig. S20** Top view RCS curves of the airplane under HP


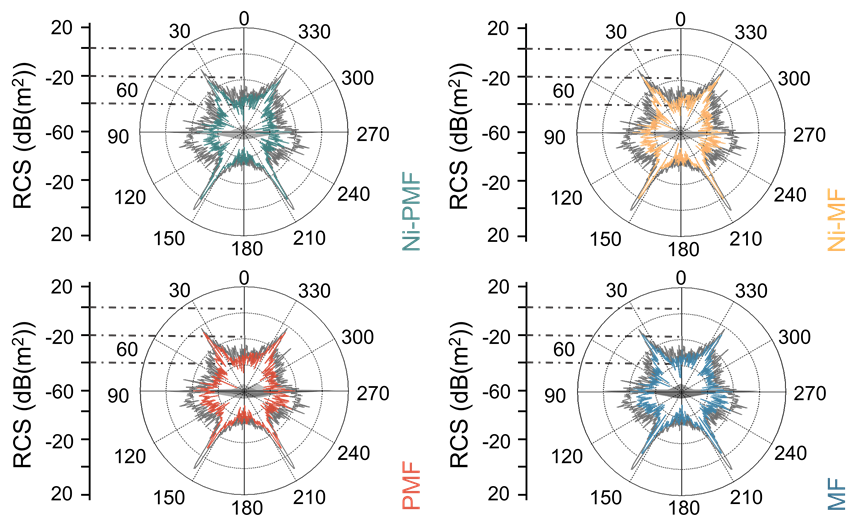


**Fig. S21** Forward view RCS curves of the airplane under VP


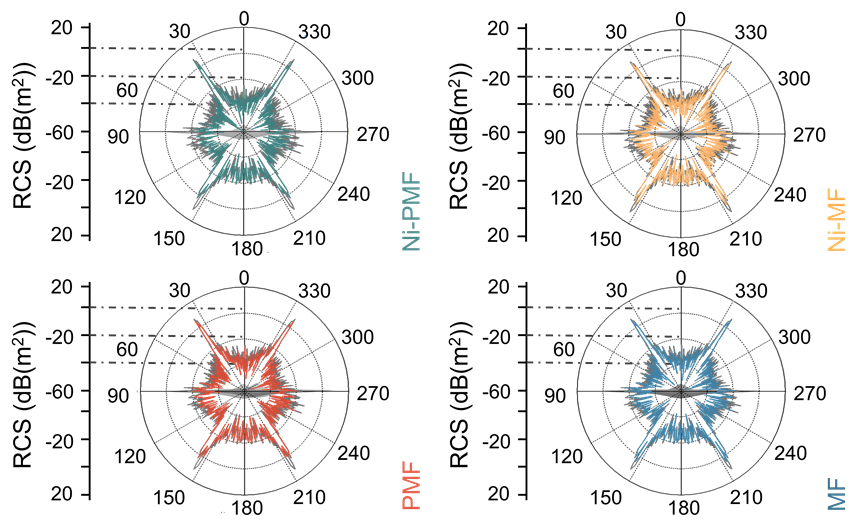


**Fig. S22** Forward view RCS curves of the airplane under HP


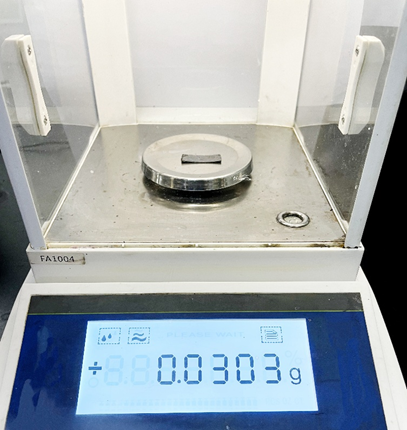


**Fig. S23** Photograph of Ni-PMF

**
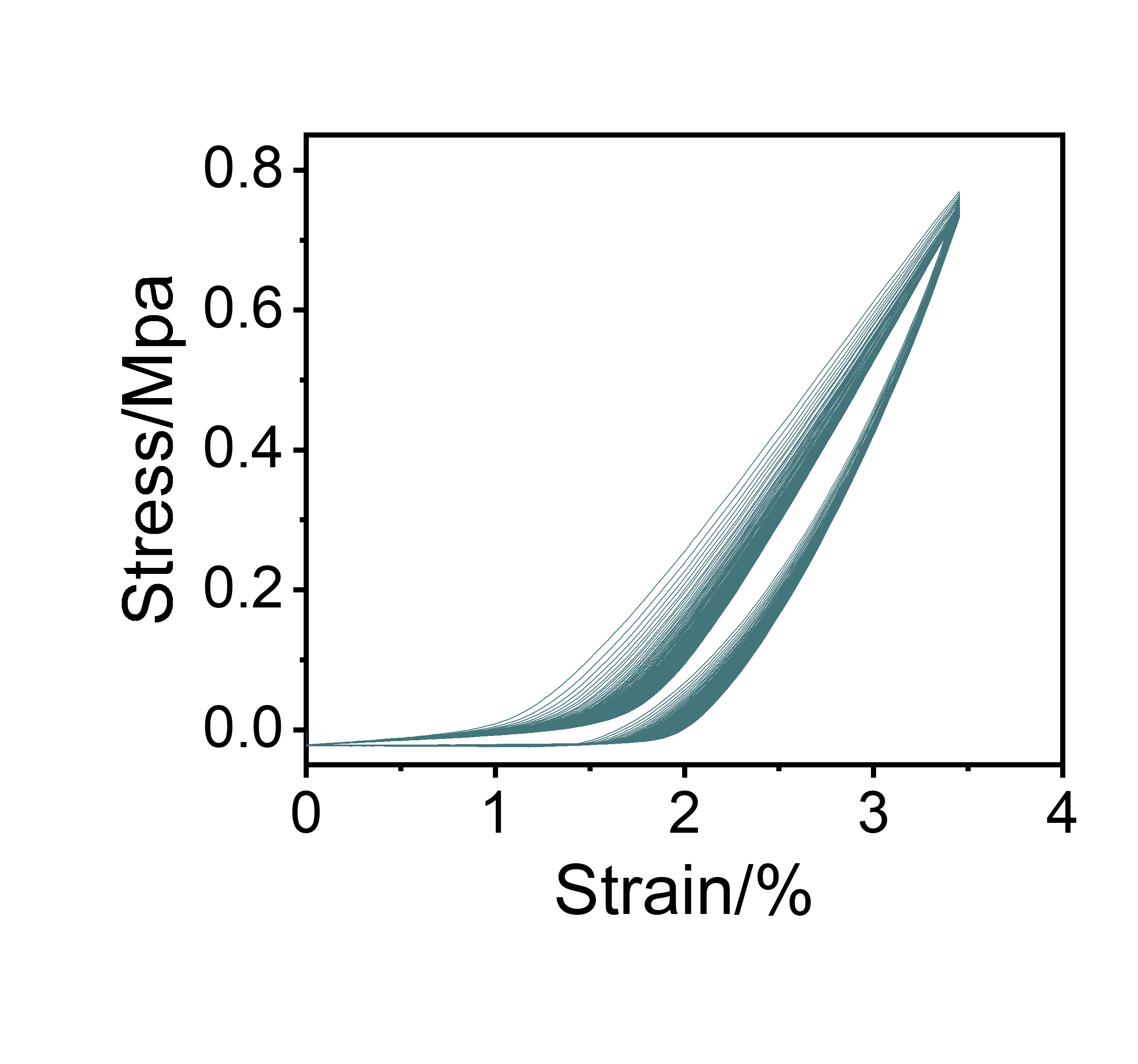
**

**Fig. S24** Tensile stress–strain curves of Ni-PMF at 50% maximum tensile strain


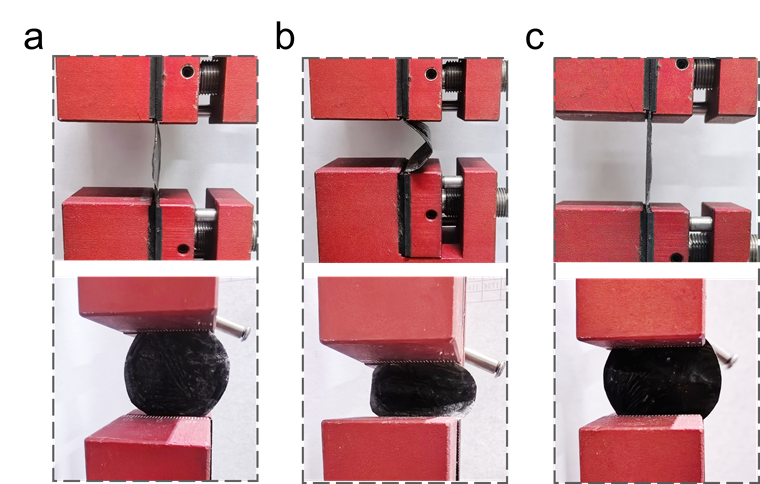


**Fig. S25** Photographs of Ni-PMF film **a** initial, **b** bending, and **c** after 100 stretching cycles


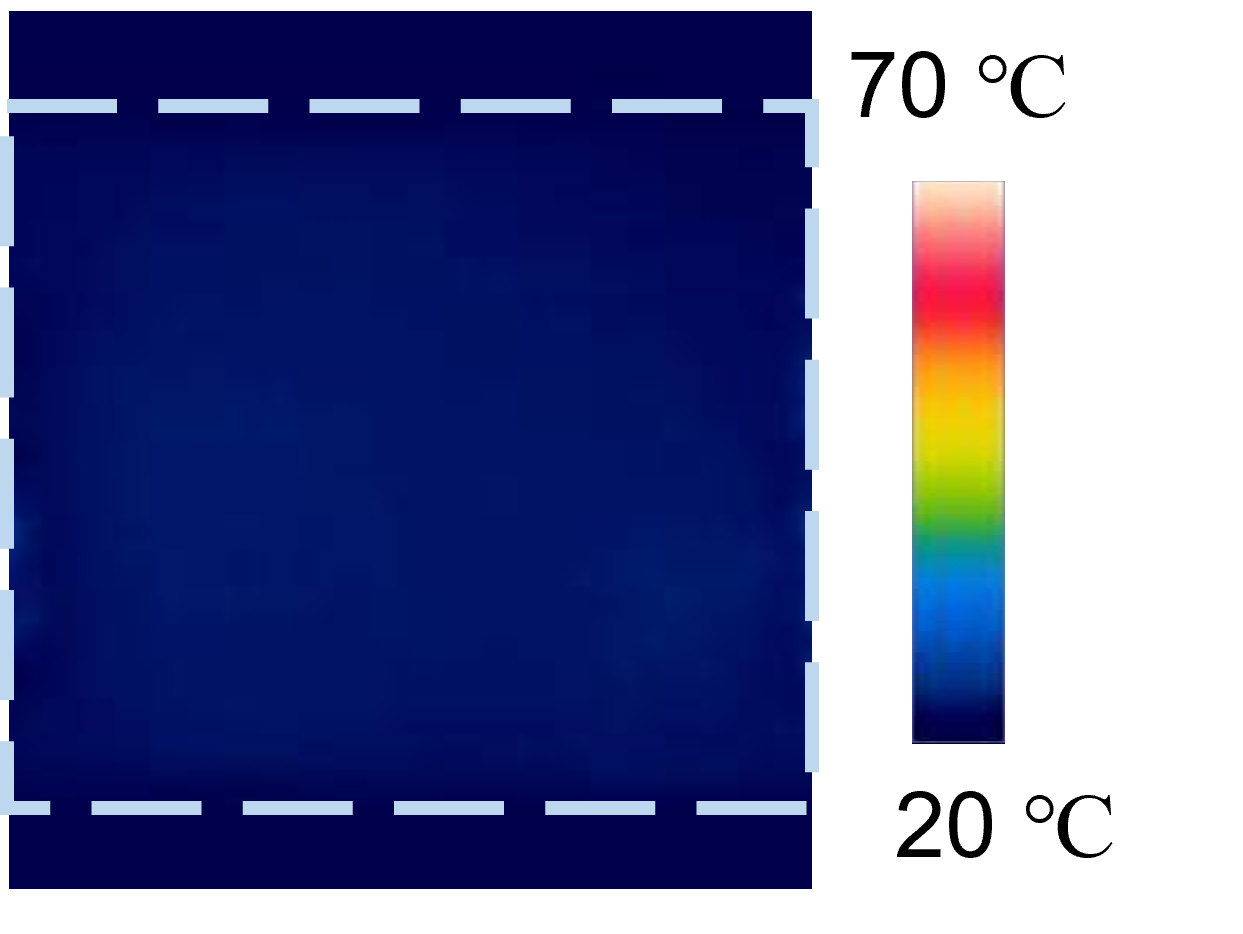


**Fig. S26** Infrared image of Ni-PMF under applied 1 V voltage


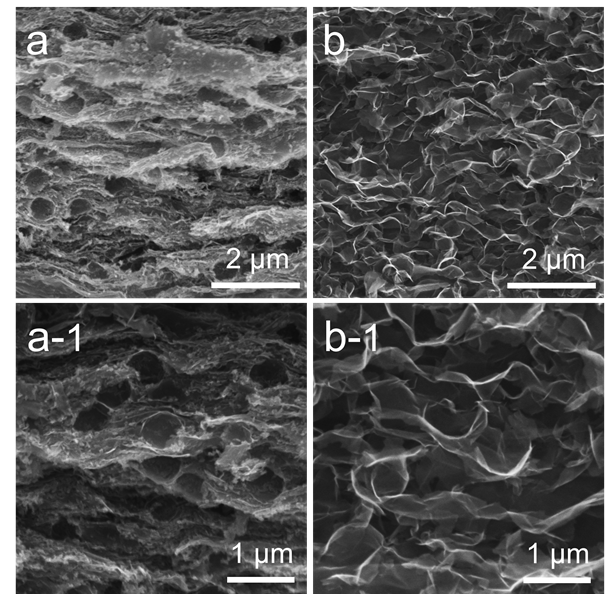


**Fig. S27** SEM images of **a** Ni-PMF-1 and **b** Ni-PMF-2
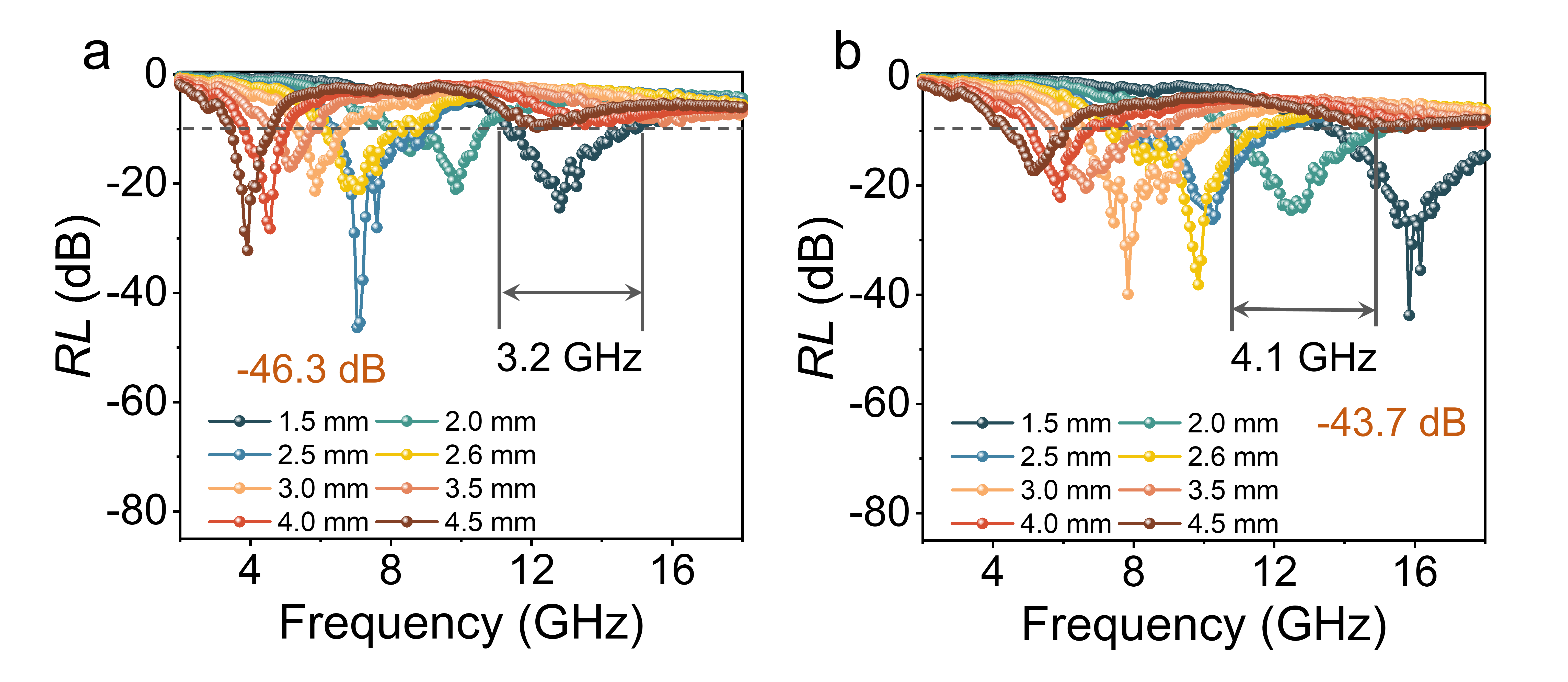


**Fig. S28** *RL* curves of **a** Ni-PMF-1 and **b** Ni-PMF-2


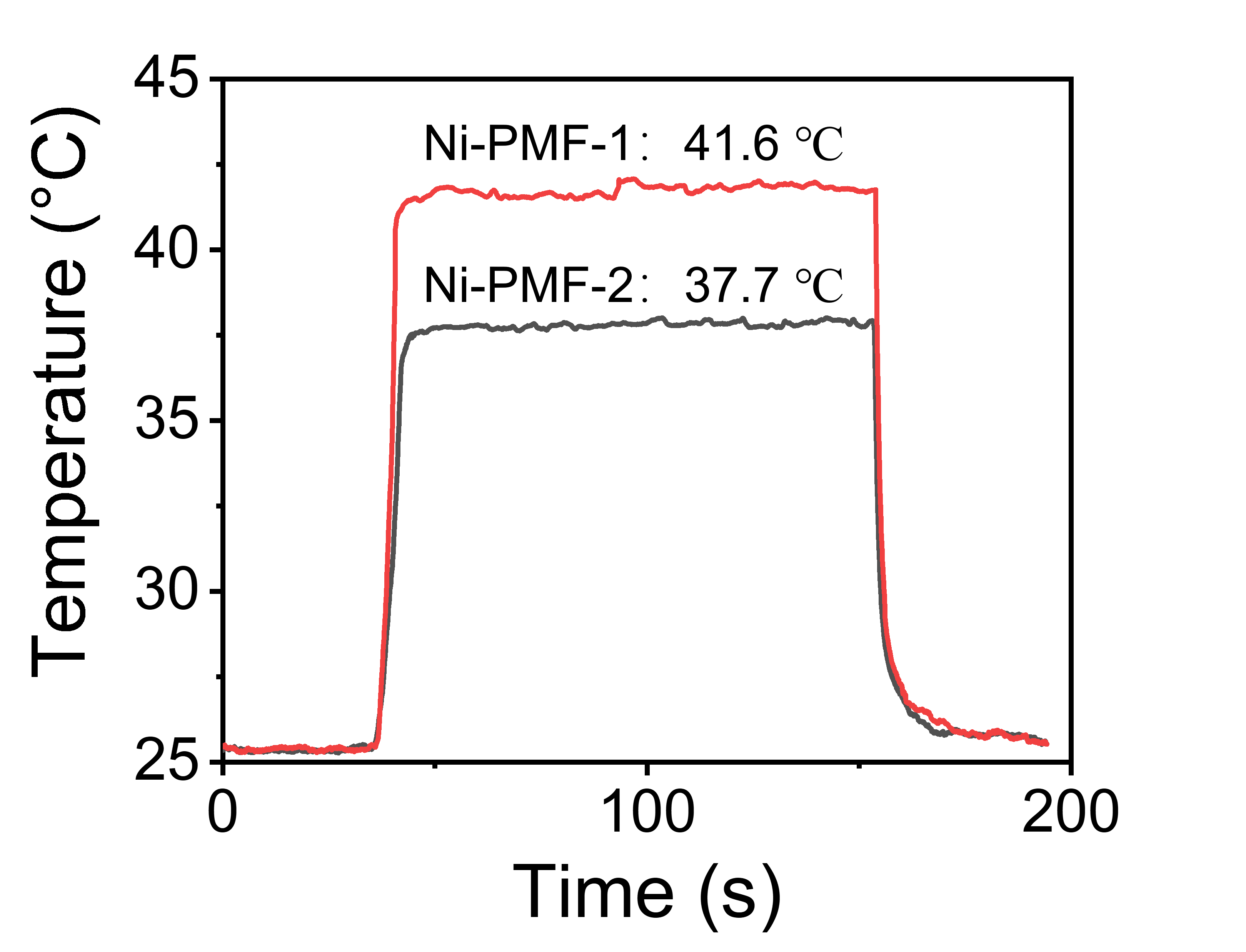


**Fig. S29** Temperature profiles of Ni-PMF-1 and Ni-PMF-2 under10 V voltage


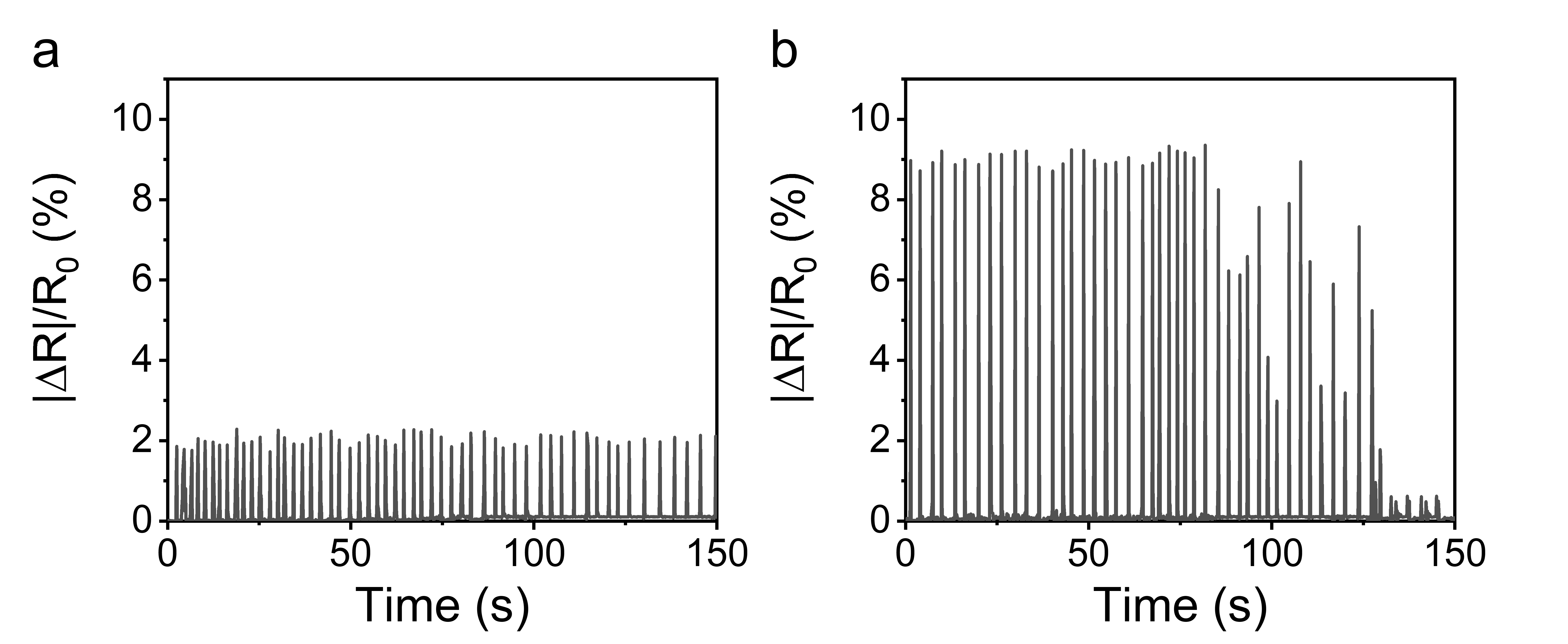


**Fig. S30** Relative resistance change of **a** Ni-PMF-1 and **b** Ni-PMF-2 under 30 ̊ bending strain


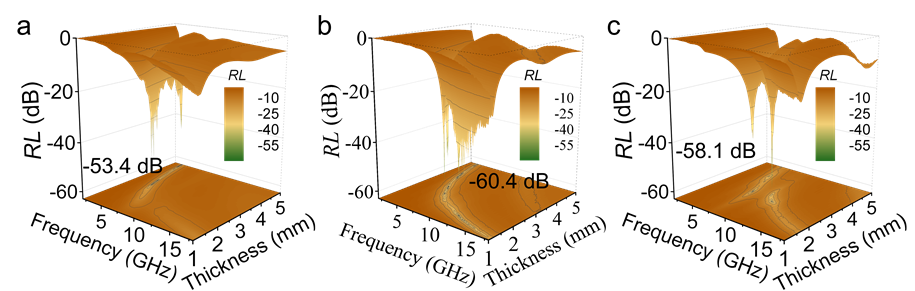


**Fig. S31** 3D *RL* curves of the Ni-PMF film: **a** after a 20 V voltage de-icing thermal management process, **b** after approximately two weeks of structural stability testing, and **c** after 80 strain sensing cycles under a bending angle of about 45°

**Table S1** XPS elemental composition ratios of the samples

| Absorber | C (%) | Ti (%) | O (%) | Ni (%) |
| --- | --- | --- | --- | --- |
| Ni-PMF | 76.46 | 6.96 | 14.64 | 1.94 |
| Ni-MF | 76.66 | 6.45 | 14.88 | 2.01 |
| PMF | 80.98 | 7.54 | 11.48 |  |
| MF | 80.37 | 7.46 | 12.17 |  |

**Table S2** Compared the EM wave absorption performance of the other reported EM wave absorbing materials

| Absorber | EAB_10_  (GHz) | *RL*  (dB) | Ref. | |
| --- | --- | --- | --- | --- |
| MrG-80 | 4.84 | -44.3 | | Carbon **208**, 374-383 (2023) |
| TCC2 | 4 | -54.8 | | Carbon **206**, 392-401 (2023) |
| CoNi/PDMS films | 1.04 | -56.7 | | Adv. Funct. Mater. **35,** 2316691 (2024) |
| CSSLS | 3.99 | -55.02 | | J. Mater. Chem. A **12**, 5377-5391 (2024) |
| CC@ZnO | 4 | -47.3 | | Compos. Part B: Eng. **229**, 109458 (2022) |
| NPC-5 | 5.92 | -63.28 | | Adv. Sci. **10**, 2304218 (2023) |
| MXene‑rGO/CoNi | 4.9 | -54.1 | | Nano-Micro Lett. **14**, 73 (2022) |
| C-GNS/ANF films | 5.28 | -56.07 | | ACS Appl. Mater. Interfaces **15**, 15872-15883 (2023) |
| SnO_2_/CFC | 5.8 | -49.1 | | Materials & Design **225**, 111576 (2023) |
| MXene/FeCo films | 0.8 | -43.7 | | Carbon **175**, 509-518 (2021) |
| MXene/C Aerogels | 5.3 | -53.02 | | Nano-Micro Lett. **15**, 194 (2023). |
| MC-2 | 5.8 | -52.6 | | Nano-Micro Lett. **16**, 212 (2024) |
| CTA-2 | 6.16 | -60.24 | | Carbon **217**, 118610 (2024) |
| Co/C/CNF/Ti_3_C_2_T_x_ | 5.86 | -62.3 | | Chem. Eng. J. **491**, 151726 (2024) |
| CNFs/MXene/Fe_3_O_4_ | 6.68 | -56.8 | | Chem. Eng. J. **475**, 146319 (2023) |
| MCLS Aerogel | 6.84 | -53.87 | | Small **20**, 2306698 (2023). |
| SL-Ti_3_C_2_T_x_ | 6.88 | -43.5 | | J. Mater. Sci. Technol. **115**, 148-155 (2022) |
| Ni-PMF | 7.2 | -64.8 | | This Work |

**Table S3** Maximum tensile strength and Young’s modulus of Ni-PMF.

| Absorber | Maximum tensile strength (MPa) | Young’s modulus (MPa) |
| --- | --- | --- |
| Ni-PMF | 1.04 | 23.54 |
